# Synthesis and Bioactivity of Hydrazide-Hydrazones with the 1-Adamantyl-Carbonyl Moiety

**DOI:** 10.3390/molecules24214000

**Published:** 2019-11-05

**Authors:** Van Hien Pham, Thi Phuong Dung Phan, Dinh Chau Phan, Binh Duong Vu

**Affiliations:** 1 Drug R&D Center, Vietnam Military Medical University. No.160, Phung Hung str., Phuc La ward, Ha Dong district, Hanoi 100000, Vietnam; phamvanhien181288@gmail.com; 2 Department of Pharmaceutical Chemistry, Hanoi University of Pharmacy. No. 15, Le Thanh Tong Str., Hoan Kiem district, Hanoi 100000, Vietnam; pdungdhd@gmail.com; 3 Hanoi University of Science and Technology. No.1, Dai Co Viet str., Bach Khoa ward, Hai Ba Trung district, Hanoi 100000, Vietnam

**Keywords:** adamantane derivatives, hydrazide-hydrazone, antimicrobial, cytotoxicity activity

## Abstract

Reaction of 1-adamantyl carbohydrazide (**1**) with various substituted benzaldehydes and acetophenones yielded the corresponding hydrazide-hydrazones with a 1-adamantane carbonyl moiety. The new synthesized compounds were tested for activities against some Gram-negative and Gram-positive bacteria, and the fungus *Candida albicans*. Compounds **4a**, **4b**, **5a**, and **5c** displayed potential antibacterial activity against tested Gram-positive bacteria and *C. albicans*, while compounds **4e** and **5e** possessed cytotoxicity against tested human cancer cell lines.

## 1. Introduction

The hydrazide-hydrazones derivatives, which play an important role in organic and medicine chemistry, have attracted a large number of researchers over the years because of their promising biological activities, including antimicrobial [1,2,3,4], anticancer [5,6,7], antituberculosis [2,8,9,10], antiviral [11], and anticonvulsant [2] activities. Some hydrazide-hydrazone components have been considered as drugs and have been used in clinics, such as nitrofurazone, furazolidone, nitrofurantoin.

Adamantane derivatives are important constituents in medicinal chemistry due to their multiple bioactivities. In the 1960s, the first adamantane derivative, amantadine, was found to have antiviral [12,13,14] activity. A number of studies were conducted, focusing on such derivatives with the hope to find new compounds and biological activities. As a result, many adamantane derivatives were discovered with numerous biological activities, such as antiviral [15,16,17,18,19,20], antimicrobial [18,20,21,22,23,24,25,26,27,28,29,30], anticancer [23,27,31,32,33,34,35], angiogenesis inhibition [36], anti-inflammation [37], 11β hydroxysteroid dehydrogenase type 1 (11β-HSD1) inhibition [38,39,40], and tyrosinase inhibition [41] activities. Among them, nine compounds were approved for use in clinics, including amantadine, memantine, rimantadine, tromantadine, adapalene, vildagliptine, saxagliptine, bromanatane, and adapromine. Therefore, the combination of two moieties, hydrazone and adamantane, had potential to confer new molecules with promising biological activities. In this study, we report the synthesis and biological activities of hydrazide-hydrazones with the 1-adamantyl carbonyl moiety.

## 2. Results and Discussion

### 2.1. Chemistry

**Scheme 1:**
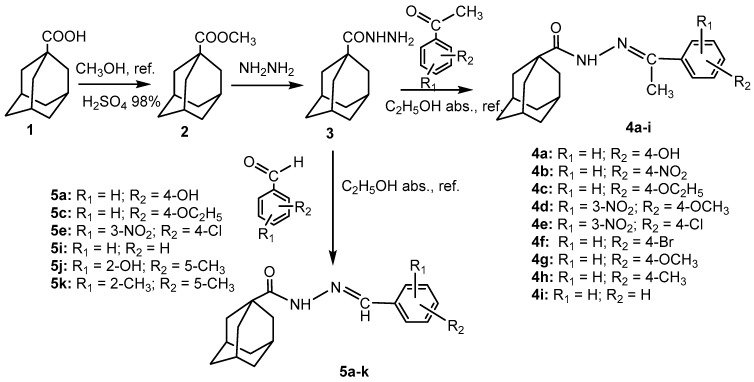
Synthesis of hydrazide-hydrazones **4a**–**i** and **5a**–**k**.

In this study, adamantane-1-carbohydrazide was used as the key intermediate. It was initially prepared by esterification of 1-adamantane carboxylic acid (**1**) and methanol under catalysis of 98% H_2_SO_4_ to yield methyl ester **2**. Then, ester **2** was reacted with hydrazine hydrate to yield adamantyl-1-carbohydrazide (**3**). Subsequently, compound **3** was condensed with aromatic aldehydes and ketones to yield the corresponding hydrazide-hydrazones **4a**–**i** and **5a**–**k**, as seen in Scheme 1 and Table 1. The structure of hydrazide hydrazones **4a**–**i** and **5a**–**k** was confirmed by ^1^H-NMR, ^13^C-NMR, and electron impact (ESI-MS) mass spectral data.

### 2.2. In Vitro Antimicrobial Activity

The newly synthesized hydrazide-hydrazones **4a**–**i** and **5a**–**k** were tested for their in vitro growth inhibition against standard strains of the National Institute for Food Control (NIFC, Vietnam), including three Gram-negative bacteria, including *Escherichia coli* (ATCC25922), *Pseudomonas aeruginosa* (ATCC27853), and *Salmonella enterica* (ATCC12228); three Gram-positive bacteria, including *Enterococcus faecalis* (ATCC13124), *Staphylococcus aureus* (ATCC25923), and *Bacillus cereus* (ATCC 13245); and one yeast-like pathogenic fungus, *Candida albicans* (ATCC10231). The primary screening was conducted using the microplate dilution method, which utilized Luria-Bertani (LB) broth medium. The antibiotic Streptomycin and antifungal drug Cyloheximide were used as the positive samples. The results of preliminary antimicrobial testing of the newly synthesized hydrazide-hydrazones **4a**–**i** and **5a**–**k** are presented in Table 2 and Table 3. 

The results of antimicrobial testing showed that all newly synthesized hydrazide-hydrazones possessed various inhibition effects on the tested Gram-positive bacteria and yeast-like fungus from moderate to serious activities. Strong antibacterial activity was found for compounds **4a**, **4d**, **5a**, and **5c**. Meanwhile, compound **4a** strongly inhibited EF, SA, BC, and CA, with an MIC of 12.5 μM (for the four strains) and IC_50_ values of 6.35 μM, 6.77 μM, 6.12 μM, and 6.37 μM, respectively. Additionally, compounds **4d**, **5a**, and **5c** have good inhibitory effects on EF and CA, with MICs of 12.5 μM and 6.26 μM, 12.5 μM and 12.5 μM, 12.5 μM and 12.5 μM; and IC_50_ values of 6.88 μM and 3.56 μM, 6.73 μM and 6.25 μM, and 6.77 μM and 6.66 μM, respectively. Moreover, compounds **4f** and **4h** showed good results in inhibition of the yeast-like fungus CA with MICs of 12.5 μM (for both synthesized compounds) and IC_50_ values of 6.77 μM and 6.45 μM, respectively. Other synthesized compounds showed moderate inhibition of the tested Gram-positive bacteria and *C. albicans*.

Regarding the structure–antimicrobial activity relationship of the synthesized compounds, inhibition increased significantly for compounds possessing an –OH (*para*) group on the benzyl ring (compounds **4a** and **5a**). Within the hydrazide-hydrazones **4a**–**i**, it was suggested that the inhibition against EF did not change substantially when having –NO_2_, –OC_2_H_5_, or an –OCH_3_ group at position 4 of the benzyl ring, but decreased if they were replaced by a –Br group. Otherwise, the presence of only one substituent on the benzyl ring inhibited SA better than that of two substituents. In the inhibition against BC, it seemed that the presence of 4-OH, 4-NO_2_, 4-OC_2_H_5_ enhanced the activity; however, it was reduced when the substituent was a 4-OCH_3_ group. In addition, the inhibition of CA showed a good result while having only one substituent of 4-NO_2_, 4-OC_2_H_5_, or 4-OCH_3_ on the benzyl ring.

In hydrazide-hydrazones **5a**–**k**, it seemed that the presence of the benzyl ring enhanced the antimicrobial activity. Compound **5k**, containing two methyl groups (2-CH_3_ and 5-CH_3_), showed good inhibition against Gram-positive bacteria and the yeast-like fungus *C. albicans*. The replacement of 2-CH_3_ by 2-OH enhanced the activity, particularly against *C. albicans*, but made no improvement to the inhibition of Gram-positive bacteria.

### 2.3. In Vitro Cytotoxicity

All the synthesized hydrazide-hydrazones **4a**–**i** and **5a**–**k** were evaluated for their cytotoxicity in four human cancer cell lines, including hepatic cancer cell line Hep3B, human cervical cancer cell line HeLa, human lung cancer cell line A549, and human breast carcinoma MCF-7, in line with a previous publication [42]. As shown in Table 4, some of the synthesized hydrazide-hydrazones exhibited antiproliferative activity against the tested cancer cell lines. Among them, compound **5e** demonstrated the most potent cytotoxicity on cell viability of 37.78 ± 2.44%, 40.42 ± 0.38%, 19.62 ± 1.74%, and 34.13 ± 2.22% in HeP3B, Hela, A549, and MCF-7 cell lines, respectively, at a concentration of 100 μM. In addition, compound **4e** inhibited Hela, A549, and MCF-7 cells, with cell viabilities of 44.37 ± 1.39%, 38.51 ± 1.59%, and 38.69 ± 1.20% at a concentration of 100 μM, respectively. Other compounds showed slight cytotoxicity on the tested cancer cell lines. Structure–cytotoxicity relationship analysis revealed that compounds **4e** and **5e**, containing both 3-NO_2_ and 4-Cl groups on the benzyl ring, have significant cytotoxicity against the four tested cancer cell lines.

## 3. Experimental

### 3.1. General

Melting point (°C) was measured in a glass microtube using a MP50 Mettler Teledo electrothermal melting point apparatus. NMR spectra were obtained with a Bruker Biospin AVANCE III spectrometer system monitoring at 500 MHz in DMSO-*d*_6_; the chemical shifts (δ, ppm) were expressed and coupling constants (J) were given in Hz using tetramethylsilane as the internal standard. Mass spectra (MS) were recorded on an Agilent 910-TQ-FT-MS system. Monitoring the reactions and preliminary purity checking of synthesized compounds were conducted by thin layer chromatography (TLC) using precoated aluminum sheet 60 F254 plates (Merck KGaA Co., Darmstadt, Germany; EMD Milipore Corp., MA, USA) and visualization with UV at 254 nm. The bacterial and fungus strains were supplied by National Institute for Food Control (NIFC, Hanoi, Vietnam) including *Escherichia coli* (ATCC25922), *Pseudomonas aeruginosa* (ATCC27853), *Salmonella enterica* (ATCC12228), *Enterococcus faecalis* (ATCC13124), *Stapphylococus aureus* (ATCC25923), *Bacillus cereus* (ATCC 13245), and *Candida albicans* (ATCC10231). This study was conducted on cancer cell lines supplied by Advanced Center for Bioorganic Chemistry (ACBC) of the Institute of Marine Biochemistry (IMBC), Vietnam Academy Science and Technology (VAST), i.e., hepatic cancer cell line Hep3B, human cervical cancer cell line HeLa, human lung cancer cell line A549, and human breast carcinoma MCF-7.

### 3.2. Synthesis of Methyl Adamantane-1-Carboxylate (***2***)

A mixture of 5 g (2.7 mmoL) adamantane-1-carboxylic acid, 50 mL methanol (1235 mmoL), and 9.2 g H_2_SO_4_ 98% was stirred under reflux conditions. The reaction was monitored by TLC performed in n-hexane/ethylacetate/dichloromethane (2/1/1, *v*/*v*/*v*) and visualized under UV at 254 nm. After the reaction, the mixture was adjusted to pH 7–8 with 10% NaHCO_3_ aqueous solution. Then, the mixture was stabilized to room temperature before 200 mL ice water was poured into it to cause precipitation. The separate precipitate was filtered, washed with water, dried, and crystallized to obtain 4.92 g of white, needle-shaped crystals with a yield of 98.4% [18].

### 3.3. Synthesis of Adamantane-1-Carbohydrazide (***3***)

A mixture of 4 g (20 mmoL) compound **2** and 25 mL (412 mmoL) 80% hydrazine hydrate aqueous solution were refluxed. The reaction was monitored by TLC using n-hexane/ethylacetate/dichloromethane (2/1/1, *v*/*v*/*v*) as the mobile phase and visualized under UV at 254 nm. At completion, 200 mL of ice water was added to the mixture. The separate precipitate was filtered and washed with water, dried to obtain 3.82 g of opalescent scaly solid with a yield of 95.5% [18].

### 3.4. Synthesis of Hydrazide-Hydrazones ***4a***–***i*** and ***5a***–***k***

A mixture of adamantane-1-carbohydrazide (**3**) and 1 mmoL of the appropriate aldehydes or ketones and 15 mL EtOH was stirred in reflux conditions. The reaction was monitored by TLC using the mobile phase as n-hexane/ethylacetate/dichloromethane (2/1/1, *v*/*v*/*v*) and visualized under UV at 254 nm. At completion, the solvent was removed under vacuum to yield a viscous liquid. It was allowed to stand at 0–5 °C to crystallize. Then, the crystal was filtered, washed with cold ethanol, and dried to obtain hydrazide-hydrazones **4a**–**i** and **5a**–**k**.

*N*′-(1-(4-hydroxyphenyl)ethylidene)adamantane-1-carbohydrazide (**4a**): M.p.: 252.5–254.1 °C, Yield 30.6%. ^1^H-NMR (500 MHz, DMSO-*d*_6_, *δ* ppm): 9.74 (1H, s, NH-N); 9.44 (1H, s, OH); 7.64 (2H, dd, *J1* = 2.0 Hz, *J2* = 7.0 Hz; Ar-H); 6.78 (2H, m, Ar-H); 2.16 (3H, s, CH_3_); 2.00 (3H, s, Adamantane-H); 1.90 (6H, s, CH_2 Ad_); 1.70 (6H, s, Adamantane-H). ^13^C-NMR (500 MHz, DMSO-*d*_6_, δ ppm): 172.6 (1C, CO); 158.8 (1C, Ar-C); 156.5 (1C, C=N); 128.9 (1C, C1); 127.9 (2C, Ar-C); 115.0 (2C, Ar-C); 38.2 (3C, Adamantane-C); 36.0 (3C, Adamantane-C); 27.6 (3C, Adamantane-C); 14.0 (1C, CH_3_). ESI-MS (*m*/*z*): [M − 1]^−1^ = 311.0; [M + 1]^+1^ = 313.1.

*N*′-(1-(4-nitrophenyl)ethylidene)adamantane-1-carbohydrazide (**4b**): M.p.: 226.0–227.6 °C; Yield 60.5%; ^1^H-NMR (500 MHz, DMSO-*d*_6_, *δ* ppm): 9.72 (1H, s, NH-N); 8.26 (2H, dd, *J1* = 2.0 Hz, *J2* = 7.0 Hz, Ar-H); 8.05 (2H, dd, *J1* = 2.0 Hz, *J2* = 7.5 Hz, Ar-H); 2.32 (3H, s, CH_3_); 2.01 (3H, s, Adamantane-H); 1.95 (6H, s, Adamantane-H); 1.71 (6H, s, Adamantane-H); ESI-MS (*m*/*z*): [M − 1]^−1^ = 340,0; [M + 1]^+1^ = 342,0.

*N*′-(1-(4-ethoxyphenyl)ethylidene)adamantane-1-carbohydrazide (**4c**): M.p.: 159.5–160.6 °C; Yield 32.2%; ^1^H-NMR (500 MHz, DMSO-*d*_6_, *δ* ppm): 9.47 (1H, s, NH-N); 7.74 (2H, d, *J* = 9.0 Hz, Ar-H); 6.95 (2H, d, *J* = 9,0 Hz, Ar-H); 4.06 (2H, dd, *J1* = 7.0 Hz; *J2* = 14.0 Hz, OCH_2_); 2.19 (3H, s, CH_3_); 2.00 (3H, s, Adamantane-H); 1.91 (6H, s, Adamantane-H); 1.70 (6H, s, Adamantane-H); 1.33 (3H, t, *J1* = 7.0 Hz, *J2* = 14.0 Hz, CH_3_). ESI-MS (*m*/*z*): [M + 1]^+1^ = 341.0.

*N*′-(1-(3-nitro-4-methoxyphenyl)ethylidene)adamantane-1-carbohydrazide (**4d**): M.p.: 182–184.1 °C; Yield 33.0%; ^1^H-NMR (500 MHz, DMSO-*d*_6_, *δ* ppm): 9.61 (1H, s, NH-N); 8.25 (1H, d, *J* = 2,5 Hz, Ar-H); 8.08 (1H, dd, *J1* = 2.0 Hz, *J2* = 8.5 Hz, Ar-H); 7.42 (1H, d, *J* = 7.0 Hz, Ar-H); 2.25 (3H, s, CH_3_); 2.00 (3H, s, Adamantane-H); 1.92 (6H, s, Adamantane-H); 1.71 (6H, s, Adamantane-H); ESI-MS (*m*/*z*): [M + 1]^+1^ = 372.0; [M − 1]^−1^ = 370.0.

*N*′-(1-(3-nitro-4-chlorophenyl)ethylidene)adamantane-1-carbohydrazide (**4e**): M.p.: 188.2–189.3 °C; Yield 26.2%. ^1^H-NMR (500 MHz, DMSO-*d*_6_, *δ* ppm): 9.72 (1H, s, NH-N); 8.39 (1H, d, *J* = 2.0 Hz, Ar-H); 8.09 (1H, dd, *J1* = 2.0 Hz, *J2* = 8.5 Hz, Ar-H); 7.83 (1H, d, *J* = 8.5 Hz, Ar-H); 2.29 (3H, s, CH_3_); 2.00 (3H, s, Adamantane-H); 1.94 (6H, s, Adamantane-H); 1.71 (6H, s, Adamantane-H). ESI-MS (*m*/*z*): [M + 1]^+1^ = 375.9; [M − 1]^−1^ = 373.9.

*N*′-(1-(3-nitro-4-chlorophenyl)ethylidene)adamantane-1-carbohydrazide (**4f**): M.p.: 190.7–191 °C. Yield: 29.0%; ^1^H-NMR (500 MHz, DMSO-*d*_6_, *δ* ppm): 9.59 (1H, s, NH-N); 7.76 (2H, d, *J* = 8.5 Hz, Ar-H); 7.63 (2H, d, *J* = 8.5 Hz, Ar-H); 2.25 (3H, s, CH_3_); 2.02 (3H, s, Adamantane-H); 1.94 (6H, s, Adamantane-H); 1.72 (6H, s, Adamantane-H); ESI-MS (*m*/*z*): [M + 1]^+1^ = 374.9.

*N*′-(1-(4-methoxyphenyl)ethylidene)adamantane-1-carbohydrazide (**4g**): M.p.: 171.6–173 °C; Yield 30.0%; ^1^H-NMR (500 MHz, DMSO-*d*_6_, *δ* ppm): 9.48 (1H, s, NH-N); 7.75 (2H, d, *J* = 8.5 Hz, Ar-H); 6.97 (2H, d, *J* = 9.0 Hz, Ar-H); 3.79 (3H, s, OCH_3_); 2.20 (3H, s, CH_3_); 2.00 (3H, s, Adamantane-H); 1.91 (6H, s, Adamantane-H); 1,70 (6H, s, Adamantane-H). ESI-MS (*m*/*z*): [M + 1]^+1^ = 327.0.

*N*′-(1-(4-methylphenyl)ethylidene)adamantane-1-carbohydrazide (**4h**): M.p.: 179.5–180.4 °C; Yield 37.3%; ^1^H-NMR (500 MHz, DMSO-*d*_6_, *δ* ppm): 9.48 (1H, s, NH-N); 7.75 (2H, d, *J* = 8.5 Hz, Ar-H); 6.97 (2H, d, *J* = 9.0 Hz, Ar-H); 3.79 (3H, s, OCH_3_); 2.20 (3H, s, CH_3_); 2.00 (3H, s, Adamantane-H); 1.91 (6H, s, Adamantane-H); 1.70 (6H, s, Adamantane-H); ESI-MS (*m*/*z*): [M + 1]^+1^ = 311.0.

*N*′-(1-phenyl)ethylidene)adamantane-1-carbohydrazide (**4i**): M.p.: 174.4–175.2 °C; Yield 54.5%; ^1^H-NMR (500 MHz, DMSO-*d*_6_, *δ* ppm): 9.54 (1H, s, NH-N); 7.79 (2H, m, Ar-H); 7.43–7.41 (3H, m, Ar-H); 2.24 (3H, s, CH_3_); 2.01 (3H, s, Adamantane-H); 1.93 (6H, s, Adamantane-H); 1.71 (6H, s, Adamantane-H). ESI-MS (*m*/*z*): [M + 1]^+1^ = 297.0.

*N*′-(4-hydroxybenzylidene)adamantane-1-carbohydrazide (**5a**): M.p.: 289.6–290.5 °C; Yield 44.0%; ^1^H-NMR (500 MHz, DMSO-*d*_6_, *δ* ppm): 10.56 (1H, s, NH-C); 9.86 (1H, s, OH); 8.28 (1H, s, N=CH); 7.49 (2H, d, *J* = 8.5 Hz, Ar-H); 6.82 (2H, d, *J* = 8.5 Hz, Ar-H); 2.01 (3H, s, Adamantane-H); 1.87 (6H, s, Adamantane-H); 1.71 (6H, s, Adamantane-H); ESI-MS (*m*/*z*): [M + 1]^+1^ = 298.9; [M − 1]^−1^ = 296.9.

*N*′-(4-ethoxybenzylidene)adamantane-1-carbohydrazide (**5c**): M.p.: 235.2–236.4 °C; Yield 15.1%; ^1^H-NMR (500 MHz, DMSO-*d*_6_, *δ* ppm): 10.61 (1H, s, NH-C); 8.32 (1H, s, N=CH); 7.57 (2H, d, *J* = 8.5 Hz, H2, H6); 6.97 (2H, d, *J* = 9.0 Hz, H3, H5); 4.07 (2H, m, OCH_2_); 2.00 (3H, s, CH_Ad_); 1.86 (6H, s, CH_2 Ad_); 1.70 (6H, s, CH_2 Ad_); 1.34 (3H, t, *J1* = 7.0 Hz, *J2* = 14.0 Hz, CH_3_); ESI-MS (*m*/*z*): [M + 1]^+1^ = 327.0.

*N*′-(3-nitro-4-chlorobenzylidene)adamantane-1-carbohydrazide (**5e**): M.p.: 247.8–248.5 °C; Yield: 50.6%; ^1^H-NMR (500 MHz, DMSO-*d*_6_, *δ* ppm): 11.12 (1H, s, NH-C); 8.43 (1H, s, N=CH); 8.29 (1H, d, J = 2.0 Hz, Ar-H); 7.96 (1H, dd, *J1* = 2.0 Hz, *J2* = 8.5 Hz, Ar-H); 7.82 (1H, d, J = 8.5 Hz, Ar-H); 2.01 (3H, s, Adamantane-H); 1.87 (6H, s, Adamantane-H); 1.70 (6H, s, Adamantane-H); ESI-MS (*m*/*z*): [M + 1]^+1^ = 361.9; [M − 1]^−1^ = 359.9.

*N*′-(benzylidene)adamantane-1-carbohydrazide (**5i**): M.p.: 186.9–187.2 °C; Yield: 60.5%; ^1^H-NMR (500 MHz, DMSO-*d*_6_, *δ* ppm): 10.77 (1H, s, NH-C); 8.40 (1H, s, N=CH); 7.66–7.61 (2H, m, Ar-H); 7.45–7.40 (3H, m, Ar-H); 2.01 (3H, s, Adamantane-H); 1.88 (6H, s, Adamantane-H); 1.70 (6H, s, Adamantane-H); ESI-MS (*m*/*z*): [M + 1]^+1^ = 283.0.

*N*′-(2-hydroxy-5-methylbenzylidene)adamantane-1-carbohydrazide (**5j**): M.p.: 247.6–248.8 °C; Yield 60.4%; ^1^H-NMR (500 MHz, DMSO-*d*_6_, *δ* ppm): 11.15 (1H, s, OH); 11.08 (1H, s, NH-C); 8.48 (1H, s, N=CH); 7.23 (1H, d, J = 1.5 Hz, Ar-H); 7.07 (1H, dd, J1 = 2.0 Hz, J2 = 8.0 Hz, Ar-H); 6.79 (1H, d, J = 8.5 Hz, Ar-H); 2.23 (3H, CH_3_); 2.01 (3H, s, Ar-H); 1.87 (6H, s, Adamantane-H); 1.70 (6H, s, Adamantane-H); ESI-MS (*m*/*z*): [M + 1]^+1^ = 313.0; [M − 1]^−1^ = 310.9.

*N*′-(2,4-diemthylbenzylidene)adamantane-1-carbohydrazide (**5k**): M.p.: 283.5–284.0 °C; Yield 35.5%; ^1^H-NMR (500 MHz, DMSO-*d*_6_, *δ* ppm): 10.72 (1H, s, NH-C); 8.63 (1H, s, N=CH); 7.59 (1H, s, H6); 7.11 (2H, s, Ar-H); 2.36 (3H, s, CH_3_); 2.29 (3H, s, CH_3_); 2.02 (3H, s, Adamantane-H); 1.88 (6H, s, Adamantane-H); 1.71 (6H, s, Adamantane-H); ESI-MS (*m*/*z*): [M + 1]^+1^ = 311.0.

Spectra of synthesized compounds were displayed in Supplemental Material file.

### 3.5. Dertemination of Antimicrobial Activity by the Dilution Method

The tested compounds **4a**–**i**, **5a**–**k** were dissolved in dimethylsulphoxide at the concentration of 100 mM, separately, to prepare the parent solutions. A total of 0.4 mL of the parent solution was withdrawn and 9.6 mL LB was added and mixed homogenously to obtain a working solution. Then, we prepared twofold serial dilution solutions in LB media at the concentrations of 100 μM, 50 μM, 25 μM, 12.5 μM, 6.25 μM, 3.13 μM, 1.56 μM, and 0.78 μM in 96 well plates, in triplicate. After that, 50 μL of microorganism suspension at 2 × 10^5^ CFU/mL was inoculated to each well and incubated at 37 °C for 24 h. Streptomycin and cycloheximide were used as the positive samples. Minimum inhibitory concentration (MIC) was determined as the lowest concentration that completely inhibited the growth of the microorganism, which was detected by the naked eye. Half of the maximal inhibitory concentration (IC_50_) is defined as the concentration of tested compounds that inhibits 50% visual growth of the test microorganism, which was determined by turbidity measurement on a Bitotech microplate reader and using RawData software [43].

### 3.6. Determination of Cytotoxicity Activity

The MTT (3-(4,5-dimethythiazol-2-yl)-2,5-diphenyl tetrazolium bromide) method was conducted to determine the cytotoxicity of the synthesized compounds **4a**–**i**, **5a**–**k** on human cancer cell lines, including hepatic cancer cell line Hep3B, human cervical cancer cell line HeLa, human lung cancer cell line A549, and human breast carcinoma MCF-7, according to a previous publication [43]. The cells, which were seeded at 5 × 10^6^ cell/well, were grown in 96-well plates with RPMI 1640 or DMEM media containing 10% fetal bovine serum (FBS), penicillin (100 IU/mL), and streptomycin (100 μg/mL) at 37 °C in a humid air incubator supplied with 5% CO_2_. The cells were allowed to grow for 24 h and then were removed to old media and treated with the sample. A total of 200 μL of the test samples was added to the well and incubated for 72 h in culture conditions. After removing the medium, 50 μL MTT solution (1 mg/mL in phosphate buffer saline) was then added to each well and the cells were further incubated at 37 °C for 4 h. After removing the MTT solution, 100 μL of isopropanol was added to each well. The absorbance was measured on a microplate reader (iMark™ Microplate Absorbance Reader, Bio-Rad Laboratories, CA, USA) at 570 nm. Suitable blank and positive controls (Camptothecine) were included. Cytotoxicity of the synthesized compounds was determined as the percent of cell survival, as follows: [OD (72 h)–OD (0 h)]/[OD(DMSO)–OD (0 h)]; where OD (72 h), OD (0 h), OD(DMSO) are the absorbance of test sample at 72 h, test sample at 0 h, and DMSO sample [42].

## 4. Conclusions

The synthesis and characterization of hydrazide-hydrazones with a 1-adamantane carbonyl moiety, **4a**–**i** and **5a**–**k**, were achieved. The synthesis was conducted by condensing adamantane-1-carbohydrazide (**3**) with substituted acetophenones to get compounds **4a**–**i**, and with substituted benzaldehydes to get compounds **5a**–**k**. The antimicrobial and cytotoxicity of the synthesized compounds were determined. The screening results revealed that compounds **4a**, **4d**, **5a**, and **5c** showed good antibacterial activity against Gram-positive bacteria and *Candida albicans* compared to known antibacterial and antifungal drugs. These compounds are considered as good candidates for new antimicrobial agents. Moreover, among the synthesized compounds, **4e** and **5e** displayed promising inhibitory activity against the growth of tested human cancer cell lines. Although the active compounds are considered to be good candidates as antimicrobial and antitumor agents, further studies, such as mechanism determination, should be undertaken.

## Figures and Tables

**Table 1:** Melting point (m.p), yield (%), molecular formulae, molecular weight (Mol. Wt.) and Rf of hydrazide-hydrazones **4a**–**i** and **5a**–**k**.

Comp. No.	R1	R2	m.p (°C)	Yield (%)	Molecular Formular (Mol. Wt.)	TLC * (Rf)
**4a**	H	4-OH	252.5–254.1	30.6	C_19_H_24_N_2_O_2_ (312.41)	0.36
**4b**	H	4-NO_2_	226.0–227.6	60.5	C_19_H_23_N_3_O_3_ (341.41)	0.58
**4c**	H	4-OC_2_H_5_	159.5–160.6	32.2	C_21_H_28_N_2_O_2_ (340.47)	0.57
**4d**	3-NO_2_	4-OCH_3_	182.0–184.1	33.0	C_20_H_25_N_3_O_4_ (371.44)	0.44
**4e**	3-NO_2_	4-Cl	188.2–189.3	26.2	C_19_H_22_ClN_3_O_3_ (375.85)	0.56
**4f**	H	4-Br	190.7–191.0	29.0	C_19_H_23_BrN_2_O (375.31)	0.62
**4g**	H	4-OCH_3_	171.6–173.0	30.0	C_20_H_26_N_2_O_2_ (326.44)	0.52
**4h**	H	4-CH_3_	179.5–180.4	37.3	C_20_H_26_N_2_O (310.44)	0.66
**4i**	H	H	174.4–175.2	54.5	C_19_H_24_N_2_O (296.41)	0.59
**5a**	H	4-OH	289.6–290.5	44.0	C_18_H_22_N_2_O_2_ (298.39)	0.33
**5c**	H	4-OC_2_H_5_	235.2–236.4	15.1	C_20_H_26_N_2_O_2_ (326.44)	0.59
**5e**	3-NO_2_	4-Cl	247.8–248.5	50.6	C_18_H_20_ClN_3_O_3_ (361.83)	0.55
**5i**	H	H	186.9–187.2	60.5	C_18_H_22_N_2_O (282.39)	0.54
**5j**	2-OH	5-CH_3_	247.6–248.8	60.4	C_19_H_24_N_2_O_2_ (312.41)	0.57
**5k**	2-CH_3_	5-CH_3_	283.5–284.0	35.5	C_20_H_26_N_2_O (310.44)	0.45

* Solvent: n-hexane/ethylacetate/dichloromethane (2/1/1, *v*/*v*/*v*), visualization at UV 254 nm.

**Table 2:** Minimum inhibitory concentration (MIC) of synthesized hydrazide-hydrazones **4a**–**i** and **5a**–**k**.

Comp. No.	MIC of Synthesized Compounds (μM)						
	Gram (+)			Gram (−)			Fungus
	EF	SA	BC	EC	PA	SE	CA
**4a**	12.5	12.5	12.5	-	-	-	12.5
**4b**	25	25	25	-	-	-	25
**4c**	25	25	25	-	-	-	25
**4d**	12.5	50	100	-	-	-	6.25
**4e**	25	50	50	-	-	-	25
**4f**	50	50	50	-	-	-	12.5
**4g**	25	25	100	-	-	-	25
**4h**	25	25	50	-	-	-	12.5
**4i**	25	50	50	-	-	-	25
**5a**	12.5	25	25	-	-	-	12.5
**5c**	12.5	50	100	-	-	-	12.5
**5e**	25	25	25	-	-	-	25
**5i**	50	50	50	-	-	-	50
**5j**	50	50	50	-	-	-	25
**5k**	25	25	25	-	-	-	25
STM	350	350	175	44	350	175	NT
CHM	NT	NT	NT	NT	NT	NT	114

EF: *Enterococcus faecalis* (ATCC13124); SA: *Staphylococcus aureus* (ATCC25923); BC: *Bacillus cereus* (ATCC 13245); EC: *Escherichia coli* (ATCC25922); PA: *Pseudomonas aeruginosa* (ATCC27853); SE: *Salmonella enterica* (ATCC12228); CA: *Candida albicans* (ATCC10231); STM: streptomycine; CHM: Cycloheximide; NT: not tested; -: inactive.

**Table 3:** IC_50_ of synthesized hydrazide-hydrazones **4a**–**i** and **5a**–**k**.

Comp. No.	IC_50_ of Synthesized Compounds (μM)						
	Gram (+)			Gram (−)			Fungus
	EF	SA	BC	EC	PA	SE	CA
**4a**	6.35	6.77	6.12	-	-	-	6.37
**4b**	11.56	11.45	12.56	-	-	-	12.78
**4c**	13.24	12.67	12.77	-	-	-	13.11
**4d**	6.88	25.45	52.11	-	-	-	3.56
**4e**	13.55	25.11	25.99	-	-	-	13.57
**4f**	24.79	13.44	25.33	-	-	-	6.77
**4g**	12.56	12.55	56.7	-	-	-	11.55
**4h**	13.22	13.45	23.88	-	-	-	6.45
**4i**	12.56	25.66	25.65	-	-	-	12.33
**5a**	6.73	12.33	12.37	-	-	-	6.25
**5c**	6.77	26.55	26.78	-	-	-	6.66
**5e**	13.25	12.67	12.33	-	-	-	13.22
**5i**	25.66	26.55	26.56	-	-	-	25.33
**5j**	24.58	24.56	24.33	-	-	-	11.45
**5k**	12.35	12.45	12.33	-	-	-	13.46

EF: *Enterococcus faecalis* (ATCC13124); SA: *Staphylococcus aureus* (ATCC25923); BC: *Bacillus cereus* (ATCC 13245); EC: *Escherichia coli* (ATCC25922); PA: *Pseudomonas aeruginosa* (ATCC27853); SE: *Salmonella enterica* (ATCC12228); CA: *Candida albicans* (ATCC10231); -: inactive.

**Table 4:** The effect of newly synthesized hydrazide-hydrazone **4a**–**i** and **5a**–**k** on the viability of HeP3B, Hela, A549, and MCF-7 cells after 48 h of incubation.

Comp. No.	Conc.	Hep3B	Hela	A549	MCF-7
**4a**	30 µM	63.89 ± 0.69	73.09 ± 2.31	67.78 ± 0.26	63.28 ± 1.41
	100 µM	56.53 ± 1.32	66.87 ± 1.10	51.63 ± 0.81	58.60 ± 0.32
**4b**	30 µM	66.83 ± 1.15	82.84 ± 1.37	66.89 ± 0.94	64.32 ± 2.92
	100 µM	56.49 ± 2.17	77.20 ± 0.90	62.26 ± 0.15	52.64 ± 2.02
**4c**	30 µM	80.62 ± 1.25	93.64 ± 0.88	77.76 ± 1.51	79.36 ± 1.40
	100 µM	72.74 ± 2.00	90.51 ± 1.28	70.20 ± 0.13	67.78 ± 0.95
**4d**	30 µM	96.80 ± 0.26	>100	65.33 ± 1.28	90.20 ± 0.25
	100 µM	84.59 ± 2.39	86.34 ± 1.35	62.01 ± 2.37	78.82 ± 2.22
**4e**	30 µM	94.59 ± 2.20	80.07 ± 1.87	64.43 ± 0.49	60.94 ± 1.39
	100 µM	55.91 ± 1.70	44.37 ± 1.39	38.51 ± 1.59	38.69 ± 1.20
**4f**	30 µM	>100	88.26 ± 1.74	76.22 ± 0.98	94.68 ± 1.33
	100 µM	97.95 ± 2.43	83.18 ± 0.20	73.68 ± 1.03	91.27 ± 2.26
**4g**	30 µM	91.63 ± 2.63	96.61 ± 1.98	87.40 ± 0.95	83.11 ± 2.86
	100 µM	77.00 ± 1.84	79.46 ± 1.28	70.61 ± 1.66	71.75 ± 1.71
**4h**	30 µM	98.29 ± 2.46	>100	78.42 ± 0.83	91.59 ± 2.29
	100 µM	75.83 ± 2.76	99.77 ± 1.89	68.99 ± 2.36	70.66 ± 2.57
**4i**	30 µM	81.96 ± 1.67	88.70 ± 1.79	68.43 ± 1.72	59.77 ± 2.41
	100 µM	80.7 ± 2.17	87.75 ± 0.29	67.73 ± 1.89	57.24 ± 0.75
**5a**	30 µM	87.03 ± 1.28	87.53 ± 0.21	69.08 ± 2.56	86.14 ± 0.49
	100 µM	68.89 ± 2.18	68.26 ± 2.02	50.78 ± 1.86	64.33 ± 1.76
**5c**	30 µM	88.96 ± 0.91	89.07 ± 1.21	90.47 ± 2.23	70.30 ± 1.23
	100 µM	78.97 ± 1.82	85.41 ± 1.34	81.32 ± 1.20	65.62 ± 0.64
**5e**	30 µM	57.77 ± 1.59	76.75 ± 1.07	36.42 ± 0.94	52.56 ± 0.75
	100 µM	37.78 ± 2.44	40.42 ± 0.38	19.62 ± 1.74	34.13 ± 2.22
**5i**	30 µM	83.16 ± 1.19	89.12 ± 2.43	79.49 ± 0.94	71.53 ± 1.64
	100 µM	74.90 ± 1.34	84.28 ± 2.12	55.22 ± 1.63	65.31 ± 1.66
**5j**	30 µM	92.80 ± 2.24	68.96 ± 2.38	78.71 ± 1.75	86.48 ± 2.08
	100 µM	85.71 ± 2.28	57.90 ± 1.35	59.18 ± 2.01	79.86 ± 2.13
**5k**	30 µM	98.51 ± 0.38	82.96 ± 0.59	62.55 ± 0.59	91.80 ± 0.35
	100 µM	83.34 ± 1.65	74.27 ± 1.67	52.39 ± 1.54	77.66 ± 1.54
CPT *	0.3 μM	69.56 ± 1.27	57.06 ± 1.35	67.68 ± 1.88	56.68 ± 0.68
	14.4 μM	37.65 ± 1.21	18.61 ± 0.56	26.74 ± 2.16	28.89 ± 1.07

* Camptothecine. Data is presented as percentage of the cell viability ± SD.

## Supplementary Materials

Supplementary materials can be found online.

## References

[1] Backes G.L., Neumann D.M., Jursic B.S. (2014). Synthesis and antifungal activity of substituted salicylaldehyde hydrazones, hydrazides and sulfohydrazides. Bioorg. Med. Chem..

[2] Küçükgüzel S.G., Mazi A., Sahin F., Öztürk S., Stables J. (2003). Synthesis and biological activities of diflunisal hydrazide–hydrazones. Eur. J. Med. Chem..

[3] Popiołek Ł., Biernasiuk A. (2017). Synthesis and investigation of antimicrobial activities of nitrofurazone analogues containing hydrazide-hydrazone moiety. Saudi Pharm. J..

[4] Rollas S., Gulerman N., Erdeniz H. (2002). Synthesis and antimicrobial activity of some new hydrazones of 4-fluorobenzoic acid hydrazide and 3-acetyl-2,5-disubstituted-1,3,4-oxadiazolines. Il Farm..

[5] Nasr T., Bondock S., Youns M. (2014). Anticancer activity of new coumarin substituted hydrazide–hydrazone derivatives. Eur. J. Med. Chem..

[6] He H., Wang X., Shi L., Yin W., Yang Z., He H., Liang Y. (2016). Synthesis, antitumor activity and mechanism of action of novel 1,3-thiazole derivatives containing hydrazide–hydrazone and carboxamide moiety. Bioorg. Med. Chem. Lett..

[7] Nasr T., Bondock S., Rashed H.M., Fayad W., Youns M., Sakr T.M. (2018). Novel hydrazide-hydrazone and amide substituted coumarin derivatives: Synthesis, cytotoxicity screening, microarray, radiolabeling and in vivo pharmacokinetic studies. Eur. J. Med. Chem..

[8] Velezheva V., Brennan P., Ivanov P., Kornienko A., Lyubimov S., Kazarian K., Nikonenko B., Majorov K., Apt A. (2016). Synthesis and antituberculosis activity of indole–pyridine derived hydrazides, hydrazide–hydrazones, and thiosemicarbazones. Bioorg. Med. Chem. Lett..

[9] Pavan F.R., Maia P.I.S., Leite S.R.A., Deflon V.M., Batista A.A., Sato D.N., Franzblau S.G., Leite C.Q.F. (2010). Thiosemicarbazones, semicarbazones, dithiocarbazates and hydrazide/hydrazones: Anti–Mycobacterium tuberculosis activity and cytotoxicity. Eur. J. Med. Chem..

[10] Bedia K.K., Elçin O., Seda U., Fatma K., Nathaly S., Sevim R., Dimoglo A. (2006). Synthesis and characterization of novel hydrazide–hydrazones and the study of their structure–antituberculosis activity. Eur. J. Med. Chem..

[11] Şenkardeş S., Kaushik-Basu N., Durmaz İ., Manvar D., Basu A., Atalay R., Küçükgüzel Ş.G. (2016). Synthesis of novel diflunisal hydrazide–hydrazones as anti-hepatitis C virus agents and hepatocellular carcinoma inhibitors. Eur. J. Med. Chem..

[12] Davies W.L., Grunert R.R., Haff R.F., McGahen J.W., Neumayer E.M., Paulshock M., Watts J.C., Wood T.R., Hermann E.C., Hoffmann C.E. (1964). Antiviral activity of 1-adamantanamine (amantadine). Science.

[13] Wendel H.A., Snyder M.T., Pell S. (1966). Trial of amantadine in epidemic influenza. Clin. Pharmacol. Ther..

[14] Vernier V.G., Harmon J.B., Stump J.M., Lynes T.E., Marvel J.P., Smith D.H. (1969). The toxicologic and pharmacologic properties of amantadine hydrochloride. Toxicol. Appl. Pharmacol..

[15] Tilley J.W., Levitan P., Kramer M.J. (1979). Adamantylthiourea derivatives as antiviral agents. J. Med. Chem..

[16] Aigami K., Inamoto Y., Takaishi N., Hattori K., Takatsuki A., Tamura G. (1975). Biologically active polycycloalkanes. 1. Antiviral adamantane derivatives. J. Med. Chem..

[17] Basarić N., Sohora M., Cindro N., Mlinarić-Majerski K., De Clercq E., Balzarini J. (2014). Antiproliferative and antiviral activity of three libraries of adamantane derivatives. Archiv Pharm..

[18] Hassan G.S., El-Emam A.A., Gad L.M., Barghash A.E.M. (2010). Synthesis, antimicrobial and antiviral testing of some new 1-adamantyl analogues. Saudi Pharm. J..

[19] Göktaş F., Vanderlinden E., Naesens L., Cesur N., Cesur Z. (2012). Microwave assisted synthesis and anti-influenza virus activity of 1-adamantyl substituted N-(1-thia-4-azaspiro[4.5]decan-4-yl)carboxamide derivatives. Bioorg. Med. Chem..

[20] El-Emam A.A., Al-Deeb O.A., Al-Omar M., Lehmann J. (2004). Synthesis, antimicrobial, and anti-HIV-1 activity of certain 5-(1-adamantyl)-2-substituted thio-1,3,4-oxadiazoles and 5-(1-adamantyl)-3-substituted aminomethyl-1,3,4-oxadiazoline-2-thiones. Bioorg. Med. Chem..

[21] Orzeszko A., Kamińska B., Orzeszko G., Starościak B.J. (2000). Synthesis and antimicrobial activity of new adamantane derivatives II. Il Farm..

[22] Orzeszko A., Gralewska R., Starościak B.J., Kazimierczuk Z. (2000). Synthesis and antimicrobial activity of new adamantane derivatives I. Acta Biochim. Pol..

[23] Wang J.J., Wang S.S., Leeb C.F., Chung M.A., Chern Y.T. (1997). In vitro antitumor and antimicrobial activities of N-substituents of maleimide by adamantane and diamantane. Chemotherapy.

[24] Al-Wahaibi L., Hassan H., Abo-Kamar A., Ghabbour H., El-Emam A. (2017). Adamantane-Isothiourea Hybrid Derivatives: Synthesis, Characterization, In Vitro Antimicrobial, and In Vivo Hypoglycemic Activities. Molecules.

[25] Balaji G.L., Sarveswari S., Vijayakumar V. (2015). Synthesis of diversely substituted adamantanes as a new class of antimicrobial agent. Res. Chem. Intermed..

[26] Al-Abdullah E., Al-Tuwaijri H., Hassan H., Al-Alshaikh M., Habib E., El-Emam A. (2015). Synthesis, antimicrobial and hypoglycemic activities of novel N-(1-adamantyl) carbothioamide derivatives. Molecules.

[27] Tabbi A., Tebbani D., Caporale A., Saturnino C., Nabavi S.F., Giuseppe P., Arra C., Canturk Z., Turan-Zitouni G., Merazig H. (2017). New Adamantyl Chalcones: Synthesis, Antimicrobial and Anticancer Activities. Curr. Top. Med. Chem..

[28] Fesatidou M., Zagaliotis P., Camoutsis C., Petrou A., Eleftheriou P., Tratrat C., Haroun M., Geronikaki A., Ciric A., Sokovic M. (2018). 5-Adamantan thiadiazole-based thiazolidinones as antimicrobial agents. Design, synthesis, molecular docking and evaluation. Bioorg. Med. Chem..

[29] El-Emam A.A., Al-Tamimi A.M.S., Al-Omar M.A., Alrashood K.A., Habib E.E. (2013). Synthesis and antimicrobial activity of novel 5-(1-adamantyl)-2-aminomethyl-4-substituted-1,2,4-triazoline-3-thiones. Eur. J. Med. Chem..

[30] El-Emam A.A., Alrashood K.A., Al-Omar M.A., Al-Tamimi A.M.S. (2012). Synthesis and antimicrobial activity of N’-heteroarylidene-1-adamantylcarbohydrazides and (+/-)-2-(1-adamantyl)-4-acetyl-5-[5-(4-substituted phenyl-3-isoxazolyl)]-1,3,4-oxadiazolines. Molecules.

[31] Aguiar D.F., Dutra L.L.A., Dantas W.M., Camelo de Carvalho G.G., Gonçalves Lemes R.P., do Ó Pessoa C., Koscky Paier C.R., Barros Araujo P.L., Araujo E.S., Pena L.J. (2019). Synthesis, Antitumor and Cytotoxic Activity of New Adamantyl O-Acylamidoximes and 3-Aryl-5-Adamantane-1, 2, 4-Oxadiazole Derivatives. Chemistry Select.

[32] Anusha S., Mohan C.D., Ananda H., Baburajeev C.P., Rangappa S., Mathai J., Fuchs J.E., Li F., Shanmugam M.K., Bender A. (2016). Adamantyl-tethered-biphenylic compounds induce apoptosis in cancer cells by targeting Bcl homologs. Bioorg. Med. Chem. Lett..

[33] Fytas C., Zoidis G., Tsotinis A., Fytas G., Khan M.A., Akhtar S., Rahman K.M., Thurston D.E. (2015). Novel 1-(2-aryl-2-adamantyl)piperazine derivatives with antiproliferative activity. Eur. J. Med. Chem..

[34] Cincinelli R., Musso L., Giannini G., Zuco V., De Cesare M., Zunino F., Dallavalle S. (2014). Influence of the adamantyl moiety on the activity of biphenylacrylohydroxamic acid-based HDAC inhibitors. Eur. J. Med. Chem..

[35] Zhu X.H., Sun J., Wang S.S., Bu W., Yao M.N., Gao K., Song Y., Zhao J.Y., Lu C.T., Zhang E.H. (2016). Synthesis, crystal structure, superoxide scavenging activity, anticancer and docking studies of novel adamantyl nitroxide derivatives. J. Mol. Struct..

[36] Luo W., Tweedie D., Beedie S.L., Vargesson N., Figg W.D., Greig N.H., Scerba M.T. (2018). Design, synthesis and biological assessment of N-adamantyl, substituted adamantyl and noradamantyl phthalimidines for nitrite, TNF-α and angiogenesis inhibitory activities. Bioorg. Med. Chem..

[37] Kadi A.A., Al-Abdullah E.S., Shehata I.A., Habib E.E., Ibrahim T.M., El-Emam A.A. (2010). Synthesis, antimicrobial and anti-inflammatory activities of novel 5-(1-adamantyl)-1,3,4-thiadiazole derivatives. Eur. J. Med. Chem..

[38] Kwon S.W., Kang S.K., Lee J.H., Bok J.H., Kim C.H., Rhee S.D., Jung W.H., Kim H.Y., Bae M.A., Song J.S. (2011). Synthesis and 11β hydroxysteroid dehydrogenase 1 inhibition of thiazolidine derivatives with an adamantyl group. Bioorg. Med. Chem. Lett..

[39] Lee Y.H., Shin Y.J., Ahn S.K. (2014). 3-Amino-N-adamantyl-3-methylbutanamide derivatives as 11β-hydroxysteroid dehydrogenase 1 inhibitor. Bioorg. Med. Chem. Lett..

[40] Tice C.M., Zhao W., Krosky P.M., Kruk B.A., Berbaum J., Johnson J.A., Bukhtiyarov Y., Panemangalore R., Scott B.B., Zhao Y. (2010). Discovery and optimization of adamantyl carbamate inhibitors of 11β-HSD1. Bioorg. Med. Chem. Lett..

[41] Baek H.S., Hong Y.D., Lee C.S., Rho H.S., Shin S.S., Park Y.H., Joo Y.H. (2012). Adamantyl N-benzylbenzamide: New series of depigmentation agents with tyrosinase inhibitory activity. Bioorg. Med. Chem. Lett..

[42] Mosmann T. (1983). Rapid colorimetric assay for cellular growth and survival: Application to proliferation and cytotoxicity assays. J. Immunol. Methods.

[43] Wiegand I., Hilpert K., Hancock R.E.W. (2008). Agar and broth dilution methods to determine the minimal inhibitory concentration (MIC) of antimicrobial substances. Nat. Protoc..

