# Supplementary material for: Synthesis and Bioactivity of Hydrazide-Hydrazones with the 1-Adamantyl-Carbonyl Moiety
Source: Molecules. 2019 Nov 5;24(21):4000. doi: 10.3390/molecules24214000 (PMC6864450; doi:10.3390/molecules24214000)

Supporting information

Article

Synthesis and Bioactivity of Hydrazide-hydrazones of 1-Adamantyl-carbonyl Moiety

Hien Pham Van[1], Dung Phan Thi Phuong[2], Chau Phan Dinh[3,*] and Duong Vu Binh[1,*]

[1] Drug R&D center, Vietnam Military Medical University. No.160, Phung Hung str., Phuc La ward, Ha Dong district, Hanoi 100000, Vietnam.

[2] Department of Pharmaceutical Chemistry, Hanoi University of Pharmacy. No. 15, Le Thanh Tong Str., Hoan Kiem district, Hanoi 100000, Vietnam.

[3] Hanoi University of Science and Technology. No.1, Dai Co Viet str., Bach Khoa ward, Hai Ba Trung district, Hanoi 100000, Vietnam.

*Correspondence: vbduong2978@gmail.com and chau.phandinh@hust.edu.vn;
Tel.: +84 983 425 460; Fax: +84 243 688 4077.

Table of content

Scheme 1. Synthesis of hydrazide hydrazones **4a-i** and **5a-k** 1
Table 1. Crystal solvent (Cryst. Solv.), melting point (m.p), yield (%), molecular formulae, molecular weight (Mol. Wt.) and Rf of hydrazide-hydrazones **4a-i** and **5a-k**. 2
Table 2. MIC of synthesized hydrazide-hydrazole **4a-i** and **5a-k** 3
Table 3. IC_{50} of synthesized hydrazide-hydrazole **4a-i** and **5a-k** 4
Table 4. The effect of newly synthesized hydrazide-hydrazole **4a-i** and **5a-k** on the viability of HeP3B, Hela, A549 and MCF-7 cell after 48 h of incubation 5
^{1}H-NMR spectrum of compound **4a** 6
^{13}C-NMR spectrum of compound **4a** 6
HMBC spectrum of compound **4a** 7
HSQC spectrum of compound **4a** 7
ESI-MS spectrum of compound **4a** (negative) 8
ESI-MS spectrum of compound **4a** (positive) 8
^{1}H-NMR spectrum of compound **4b** 9
ESI-MS spectrum of compound **4b** (negative) 9
ESI-MS spectrum of compound **4b** (positive) 10
^{1}H-NMR spectrum of compound **4c** 10
ESI-MS spectrum of compound **4c** (negative) 11
^{1}H-NMR spectrum of compound **4d** 11
ESI-MS spectrum of compound **4d** (negative) 12
ESI-MS spectrum of compound **4d** (positive) 12
^{1}H-NMR spectrum of compound **4e** 13
ESI-MS spectrum of compound **4e** (negative) 13
ESI-MS spectrum of compound **4e** (positive) 14
^{1}H-NMR spectrum of compound **4f** 14
ESI-MS spectrum of compound **4f** (positive) 14
^{1}H-NMR spectrum of compound **4g** 15
ESI-MS spectrum of compound **4g** (positive) 15
^{1}H-NMR spectrum of compound **4h** 16
ESI-MS spectrum of compound **4h** (positive) 16
^{1}H-NMR spectrum of compound **4i** 17
ESI-MS spectrum of compound **4i** (positive) 18
^{1}H-NMR spectrum of compound **5a** 18
ESI-MS spectrum of compound **5a** (negative) 18
ESI-MS spectrum of compound **5a** (positive) 19
^{1}H-NMR spectrum of compound **5c** 20
ESI-MS spectrum of compound **5c** (positive) 20
^{1}H-NMR spectrum of compound **5e** 21
ESI-MS spectrum of compound **5e** (negative) 21
ESI-MS spectrum of compound **5e** (positive) 22
^{1}H-NMR spectrum of compound **5i** 22
ESI-MS spectrum of compound **5i** (positive) 22
^{1}H-NMR spectrum of compound **5j** 23
ESI-MS spectrum of compound **5j** (negative) 23
ESI-MS spectrum of compound **5j** (positive) 24
^{1}H-NMR spectrum of compound **5k** 24
ESI-MS spectrum of compound **5k** (positive) 25

Scheme 1. Synthesis of hydrazide hydrazones **4a-i** and **5a-k**

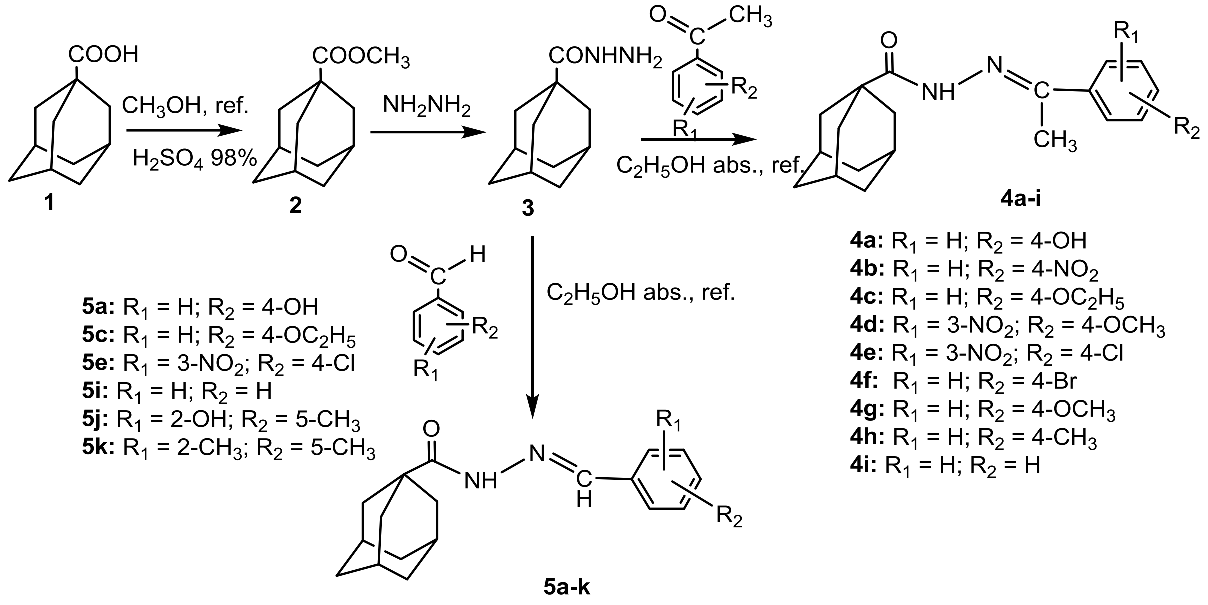

Table 1. Crystal solvent (Cryst. Solv.), melting point (m.p), yield (%), molecular formulae, molecular weight (Mol. Wt.) and Rf of hydrazide-hydrazones **4a-i** and **5a-k**.

Comp. No.	R1	R2	Cryst. Solv.	m.p (◦C)	Yield (%)	Molecular Formular (Mol. Wt.)	TLC* (Rf)
4a	H	4-OH	EtOH	252.5-254.1	30.6	$C_{19}H_{24}N_2O_2$ (312.41)	0.36
4b	H	4-NO2	EtOH	226.0-227.6	60.5	$C_{19}H_{23}N_3O_3$ (341.41)	0.58
4c	H	4-OC2H5	EtOH	159.5-160.6	32.2	$C_{21}H_{28}N_2O_2$ (340.47)	0.57
4d	3-NO2	4-OCH3	EtOH	182.0-184.1	33.0	$C_{20}H_{25}N_3O_4$ (371.44)	0.44
4e	3-NO2	4-Cl	EtOH	188.2-189.3	26.2	$C_{19}H_{22}ClN_3O_3$ (375.85)	0.56
4f	H	4-Br	EtOH	190.7-191.0	29.0	$C_{19}H_{23}BrN_2O$ (375.31)	0.62
4g	H	4-OCH3	EtOH	171.6-173.0	30.0	$C_{20}H_{26}N_2O_2$ (326.44)	0.52
4h	H	4-CH3	EtOH	179.5-180.4	37.3	$C_{20}H_{26}N_2O$ (310.44)	0.66
4i	H	H	EtOH	174.4-175.2	54.5	$C_{19}H_{24}N_2O$ (296.41)	0.59
5a	H	4-OH	EtOH	289.6 -290.5	44.0	$C_{18}H_{22}N_2O_2$ (298.39)	0.33
5c	H	4-OC2H5	EtOH	235.2-236.4	15.1	$C_{20}H_{26}N_2O_2$ (326.44)	0.59
5e	3-NO2	4-Cl	EtOH	247.8-248.5	50.6	$C_{18}H_{20}ClN_3O_3$ (361.83)	0.55
5i	H	H	EtOH	186.9-187.2	60.5	$C_{18}H_{22}N_2O$ (282.39)	0.54
5j	2-OH	5-CH3	EtOH	247.6-248.8	60.4	$C_{19}H_{24}N_2O_2$ (312.41)	0.57
5k	2-CH3	5-CH3	EtOH	283.5-284.0	35.5	$C_{20}H_{26}N_2O$ (310.44)	0.45

Table 2. MIC of synthesized hydrazide-hydrazole **4a-i** and **5a-k**

Comp. No.	MIC of synthesized compounds (µM)						
	Gram (+)			Gram (-)			Fungus
	**EF**	**SA**	**BC**	**EC**	**PA**	**SE**	**CA**
**4a**	12.5	12.5	12.5	-	-	-	12.5
**4b**	25	25	25	-	-	-	25
**4c**	25	25	25	-	-	-	25
**4d**	12.5	50	100	-	-	-	6.25
**4e**	25	50	50	-	-	-	25
**4f**	50	50	50	-	-	-	12.5
**4g**	25	25	100	-	-	-	25
**4h**	25	25	50	-	-	-	12.5
**4i**	25	50	50	-	-	-	25
**5a**	12.5	25	25	-	-	-	12.5
**5c**	12.5	50	100	-	-	-	12.5
**5e**	25	25	25	-	-	-	25
**5i**	50	50	50	-	-	-	50
**5j**	50	50	50	-	-	-	25
**5k**	25	25	25	-	-	-	25
STM	256 µg/mL	256 µg/mL	128 µg/mL	32 µg/mL	256 µg/mL	128 µg/mL	NT
CHM	NT	NT	NT	NT	NT	NT	32 µg/mL

EF: Enterococcus faecalis (ATCC13124); SA: *Stapphylococus aureus* (ATCC25923); BC: *Bacillus cereus* (ATCC 13245); EC: *Escherichia coli* (ATCC25922); PA: *Pseudomonas aeruginosa* (ATCC27853); SE: *Salmonella enterica* (ATCC12228); CA: *Candida albicans* (ATCC10231); STM: streptomycine; CHM: Cycloheximide; NT: not tested; - : inactive

Table 3. IC_{50} of synthesized hydrazide-hydrazole **4a-i** and **5a-k**

**Comp. No.**	**IC50 of synthesized compounds (μM)**						
	**Gram (+)**			**Gram (-)**			**Fungus**
	**EF**	**SA**	**BC**	**EC**	**PA**	**SE**	**CA**
**4a**	6.35	6.77	6.12	-	-	-	6.37
**4b**	11.56	11.45	12.56	-	-	-	12.78
**4c**	13.24	12.67	12.77	-	-	-	13.11
**4d**	6.88	25.45	52.11	-	-	-	3.56
**4e**	13.55	25.11	25.99	-	-	-	13.57
**4f**	24.79	13.44	25.33	-	-	-	6.77
**4g**	12.56	12.55	56.7	-	-	-	11.55
**4h**	13.22	13.45	23.88	-	-	-	6.45
**4i**	12.56	25.66	25.65	-	-	-	12.33
**5a**	6.73	12.33	12.37	-	-	-	6.25
**5c**	6.77	26.55	26.78	-	-	-	6.66
**5e**	13.25	12.67	12.33	-	-	-	13.22
**5i**	25.66	26.55	26.56	-	-	-	25.33
**5j**	24.58	24.56	24.33	-	-	-	11.45
**5k**	12.35	12.45	12.33	-	-	-	13.46

EF: *Enterococcus faecalis* (ATCC13124); SA: *Stapphylococus aureus* (ATCC25923); BC: *Bacillus cereus* (ATCC 13245); EC: *Escherichia coli* (ATCC25922); PA: *Pseudomonas aeruginosa* (ATCC27853); SE: *Salmonella enterica* (ATCC12228); CA: *Candida albicans* (ATCC10231); - : inactive.

Table 4. The effect of newly synthesized hydrazide-hydrazole **4a-i** and **5a-k** on the viability of HeP3B, Hela, A549 and MCF-7 cell after 48 h of incubation

Comp. No.	Conc.	Hep3B	Hela	A549	MCF-7
**4a**	30µM	63.89 ± 0.69	73.09 ± 2.31	67.78 ± 0.26	63.28 ± 1.41
	100 µM	56.53 ± 1.32	66.87 ± 1.10	51.63 ± 0.81	58.60 ± 0.32
**4b**	30µM	66.83 ± 1.15	82.84 ± 1.37	66.89 ± 0.94	64.32 ± 2.92
	100 µM	56.49 ± 2.17	77.20 ± 0.90	62.26 ± 0.15	52.64 ± 2.02
**4c**	30µM	80.62 ± 1.25	93.64 ± 0.88	77.76 ± 1.51	79.36 ± 1.40
	100 µM	72.74 ± 2.00	90.51 ± 1.28	70.20 ± 0.13	67.78 ± 0.95
**4d**	30µM	96.80 ± 0.26	> 100	65.33 ± 1.28	90.20 ± 0.25
	100 µM	84.59 ± 2.39	86.34 ± 1.35	62.01 ± 2.37	78.82 ± 2.22
**4e**	30µM	94.59 ± 2.20	80.07 ± 1.87	64.43 ± 0.49	60.94 ± 1.39
	100 µM	55.91 ± 1.70	44.37 ± 1.39	38.51 ± 1.59	38.69 ± 1.20
**4f**	30µM	>100	88.26 ± 1.74	76.22 ± 0.98	94.68 ± 1.33
	100 µM	97.95 ± 2.43	83.18 ± 0.20	73.68 ± 1.03	91.27 ± 2.26
**4g**	30µM	91.63 ± 2.63	96.61 ± 1.98	87.40 ± 0.95	83.11 ± 2.86
	100 µM	77.00 ± 1.84	79.46 ± 1.28	70.61 ± 1.66	71.75 ± 1.71
**4h**	30µM	98.29 ± 2.46	> 100	78.42 ± 0.83	91.59 ± 2.29
	100 µM	75.83 ± 2.76	99.77 ± 1.89	68.99 ± 2.36	70.66 ± 2.57
**4i**	30µM	81.96 ± 1.67	88.70 ± 1.79	68.43 ± 1.72	59.77 ± 2.41
	100 µM	80.7 ± 2.17	87.75 ± 0.29	67.73 ± 1.89	57.24 ± 0.75
**5a**	30µM	87.03 ± 1.28	87.53 ± 0.21	69.08 ± 2.56	86.14 ± 0.49
	100 µM	68.89 ± 2.18	68.26 ± 2.02	50.78 ± 1.86	64.33 ± 1.76
**5c**	30µM	88.96 ± 0.91	89.07 ± 1.21	90.47 ± 2.23	70.30 ± 1.23
	100 µM	78.97 ± 1.82	85.41 ± 1.34	81.32 ± 1.20	65.62 ± 0.64
**5e**	30µM	57.77 ± 1.59	76.75 ± 1.07	36.42 ± 0.94	52.56 ± 0.75
	100 µM	37.78 ± 2.44	40.42 ± 0.38	19.62 ± 1.74	34.13 ± 2.22
**5i**	30µM	83.16 ± 1.19	89.12 ± 2.43	79.49 ± 0.94	71.53 ± 1.64
	100 µM	74.90 ± 1.34	84.28 ± 2.12	55.22 ± 1.63	65.31 ± 1.66
**5j**	30µM	92.80 ± 2.24	68.96 ± 2.38	78.71 ± 1.75	86.48 ± 2.08
	100 µM	85.71 ± 2.28	57.90 ± 1.35	59.18 ± 2.01	79.86 ± 2.13
**5k**	30µM	98.51 ± 0.38	82.96 ± 0.59	62.55 ± 0.59	91.80 ± 0.35
	100 µM	83.34 ± 1.65	74.27 ± 1.67	52.39 ± 1.54	77.66 ± 1.54
**CPT***	0.1µg/mL	69.56 ± 1.27	57.06 ± 1.35	67.68 ± 1.88	56.68 ± 0.68
	5 µg/mL	37.65 ± 1.21	18.61 ± 0.56	26.74 ± 2.16	28.89 ± 1.07

*Camptothecine.
Data is presented as percentage of the cell viability ± SD.

^{1}H-NMR spectrum of compound **4a**

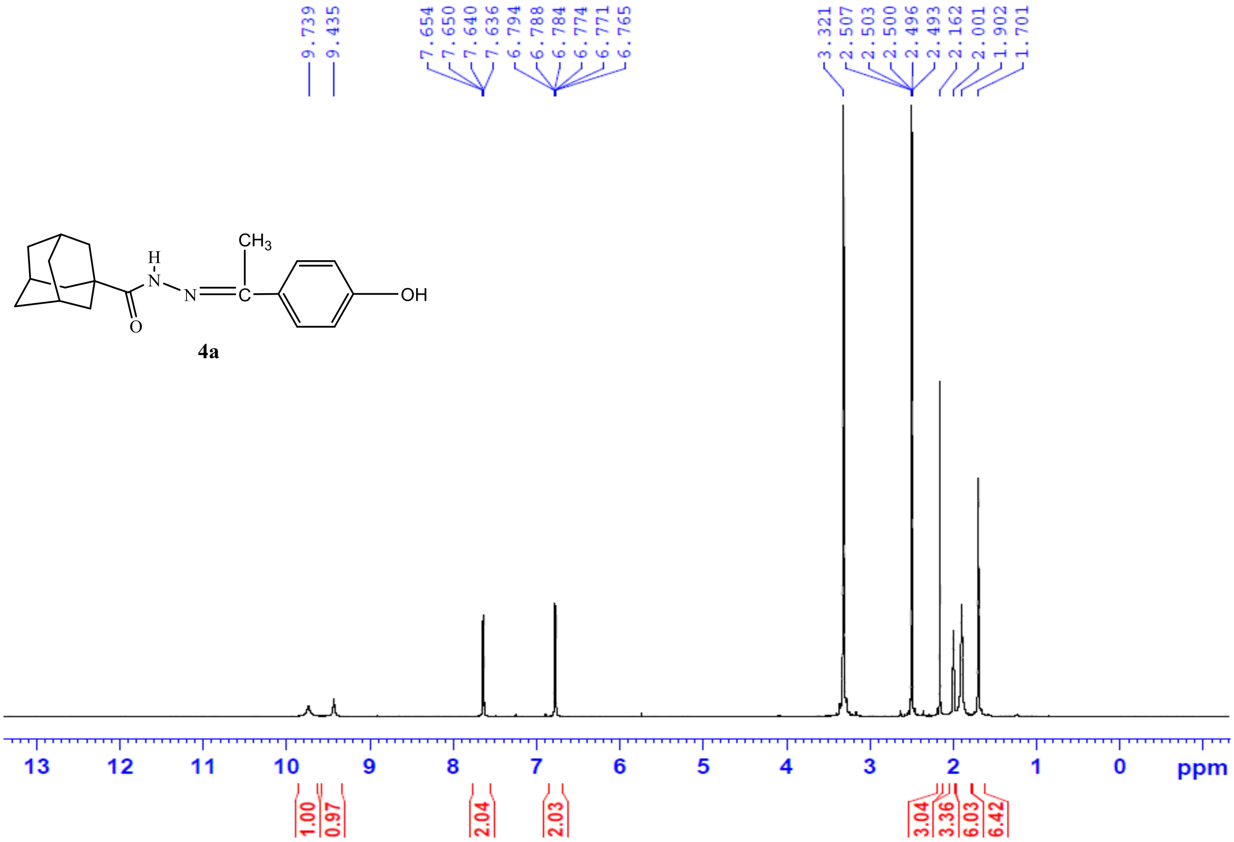

^{13}C-NMR spectrum of compound **4a**

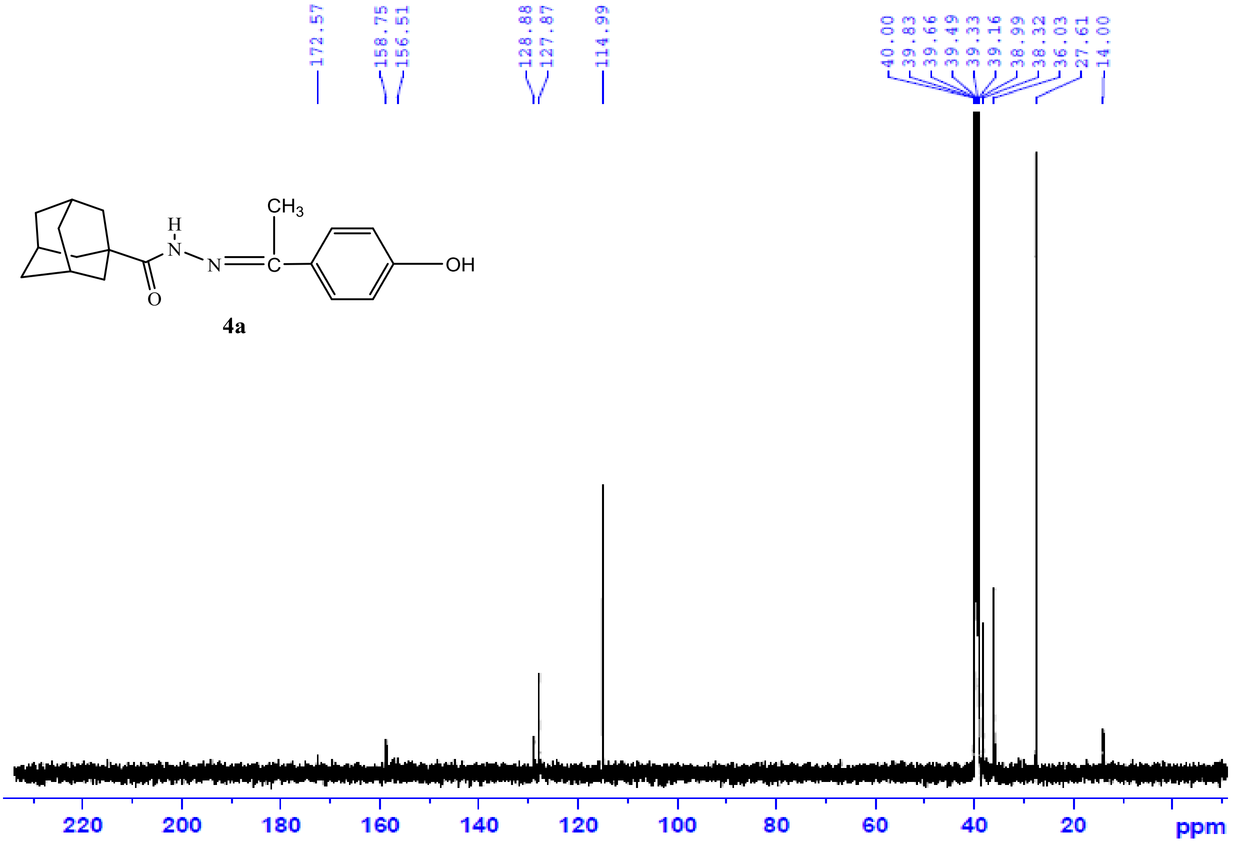

HMBC spectrum of compound **4a**

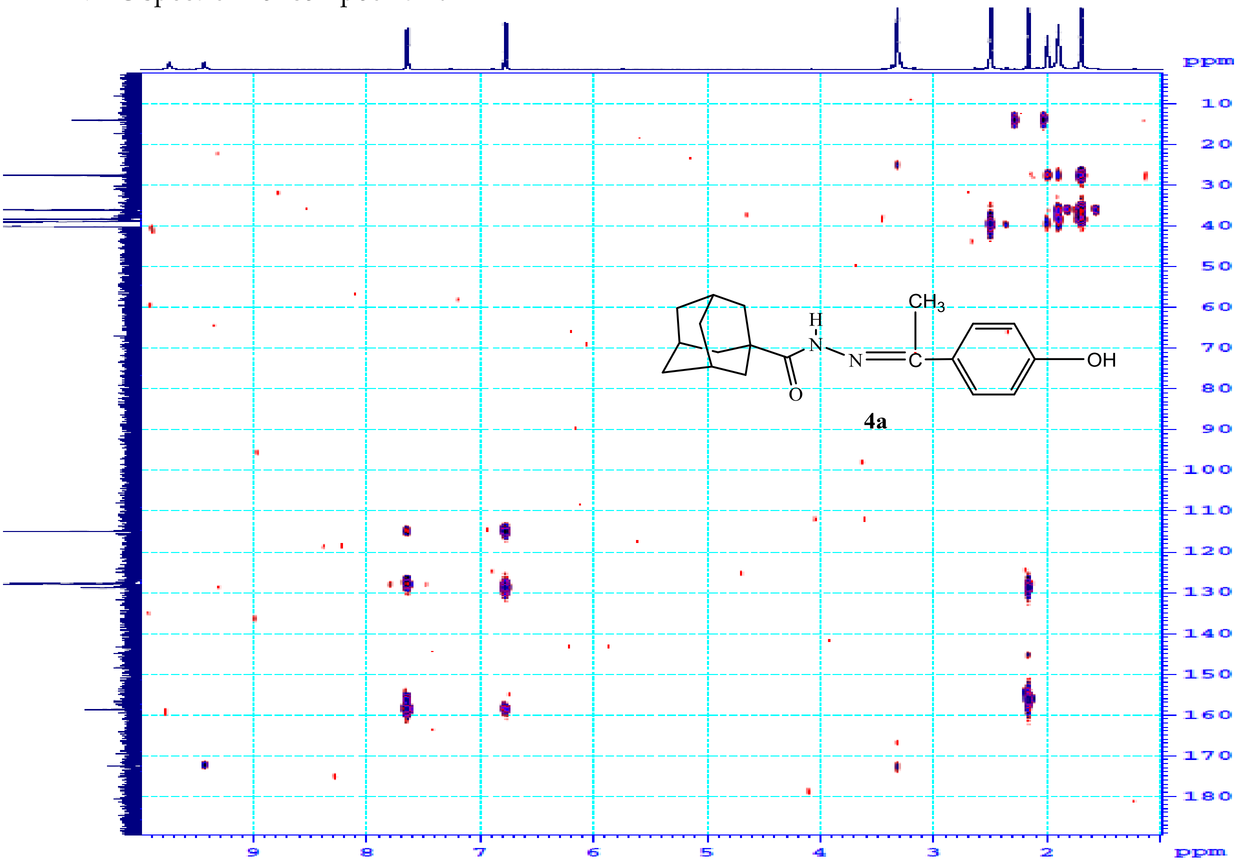

HSQC spectrum of compound **4a**

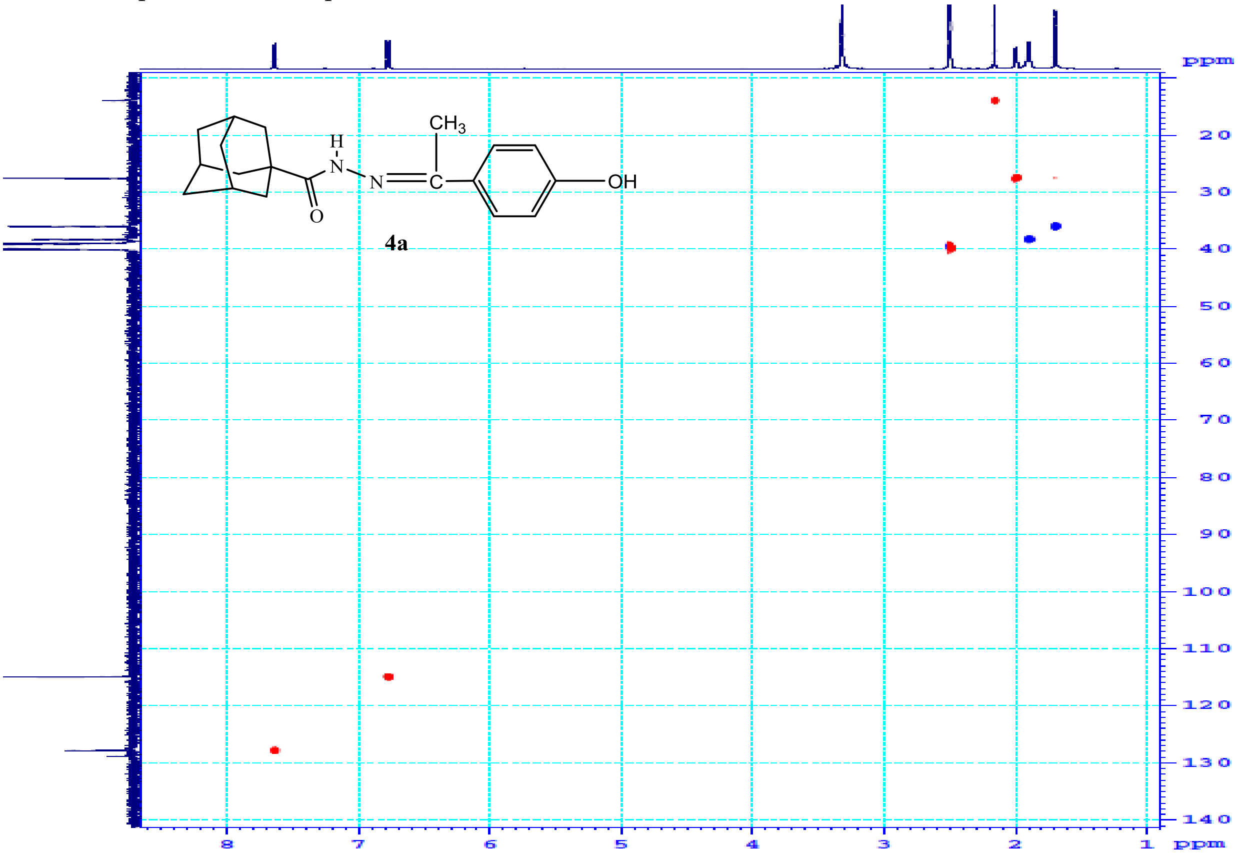

ESI-MS spectrum of compound **4a** (negative)

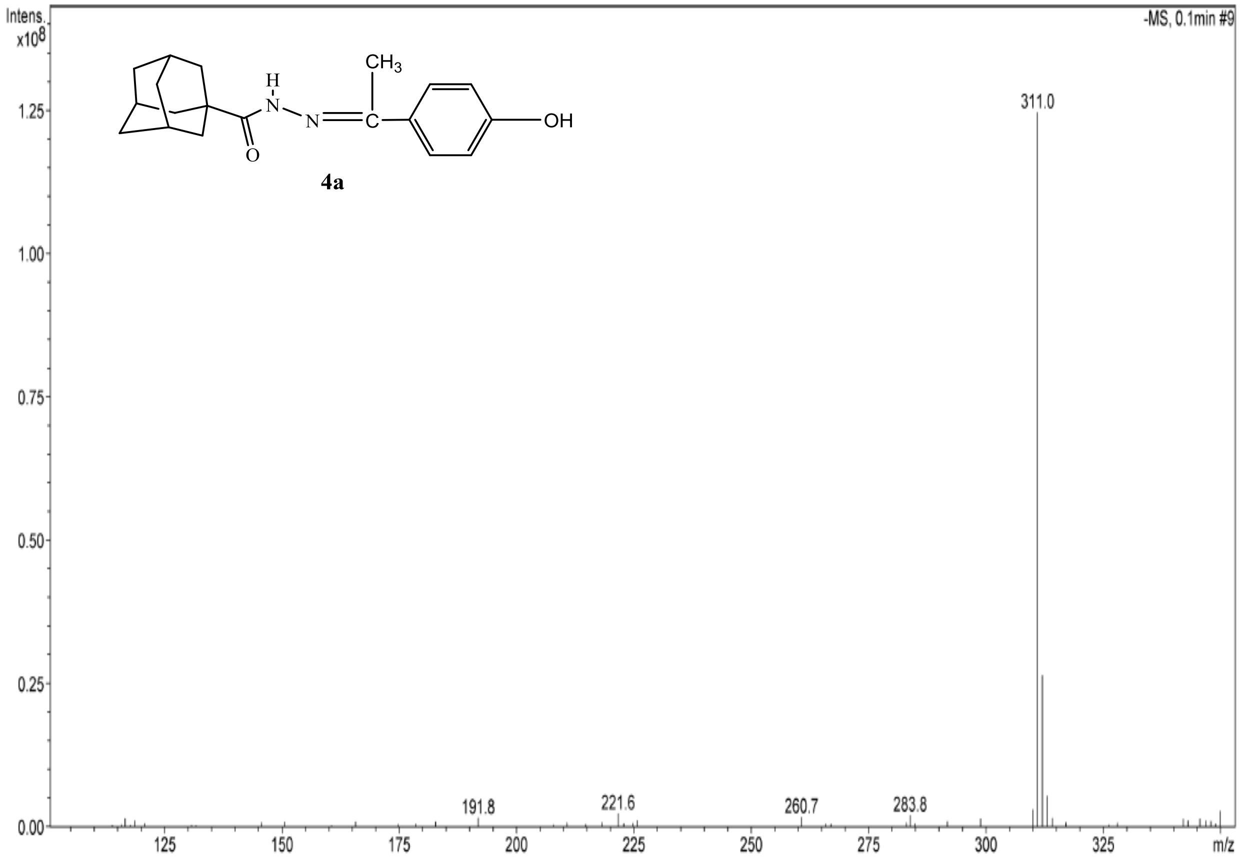

ESI-MS spectrum of compound **4a** (positive)

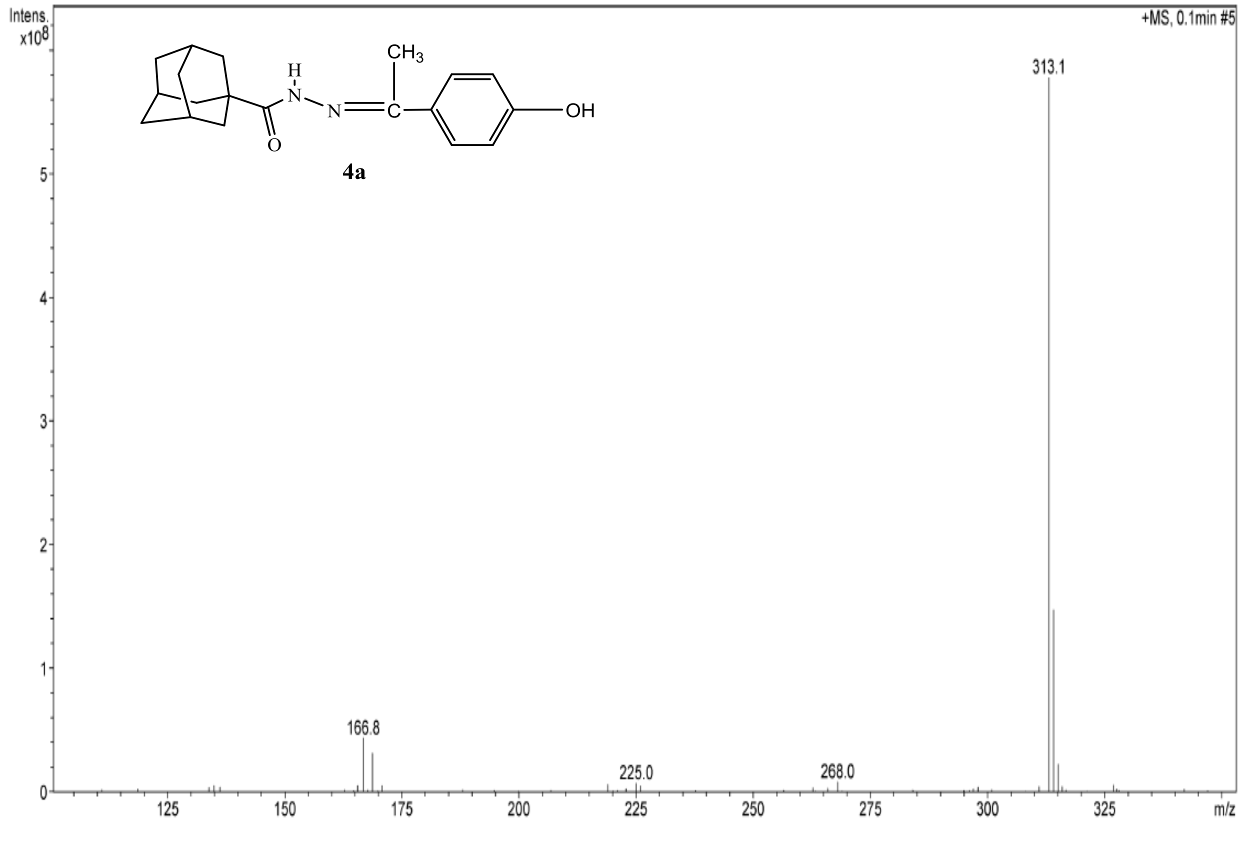

^{1}H-NMR spectrum of compound **4b**

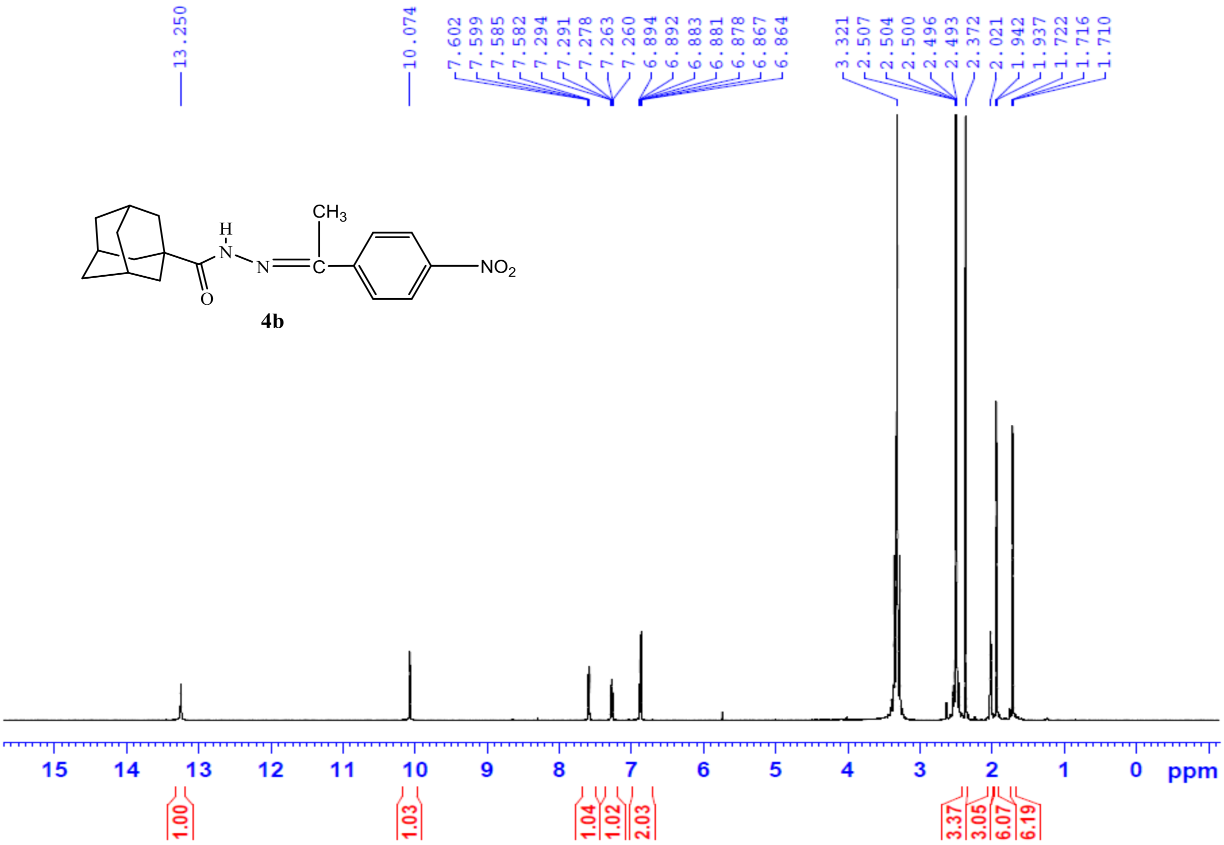

ESI-MS spectrum of compound **4b** (negative)

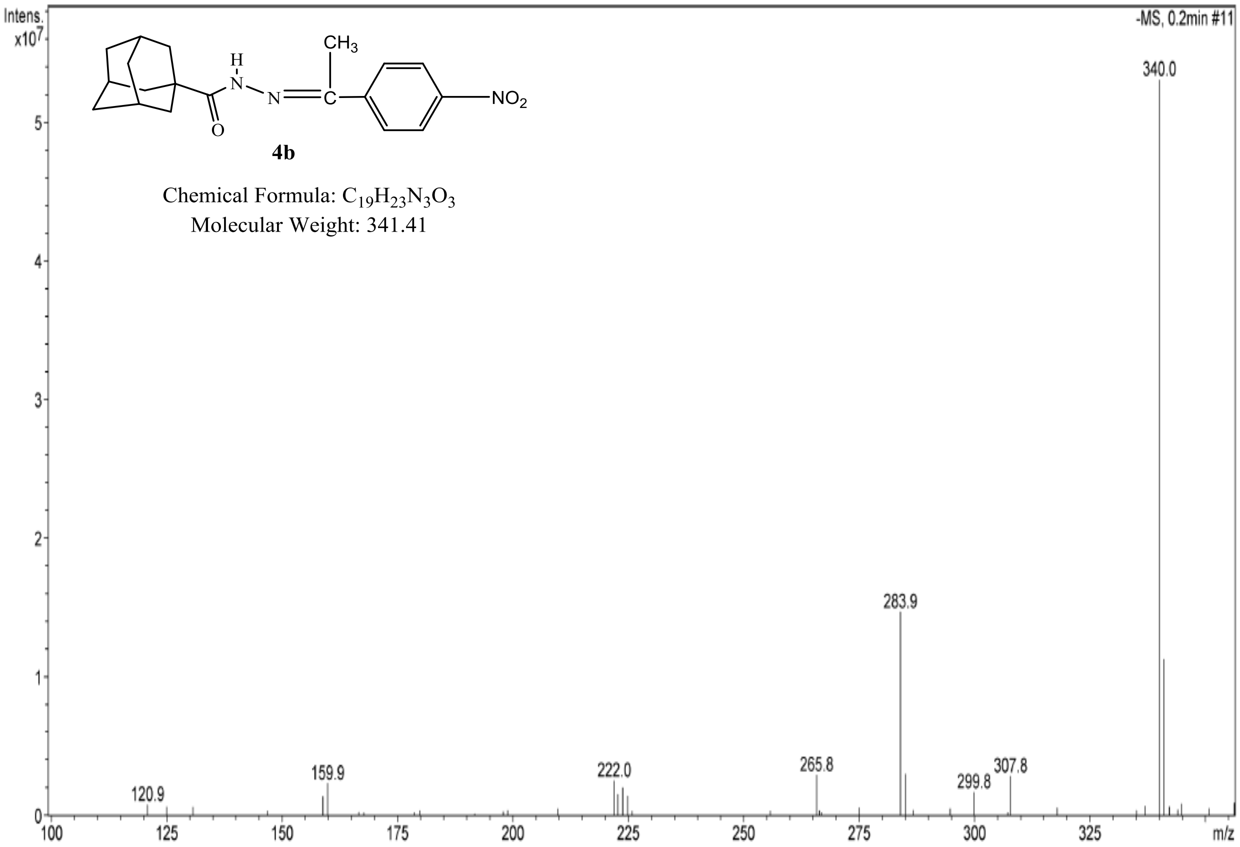

ESI-MS spectrum of compound **4b** (positive)

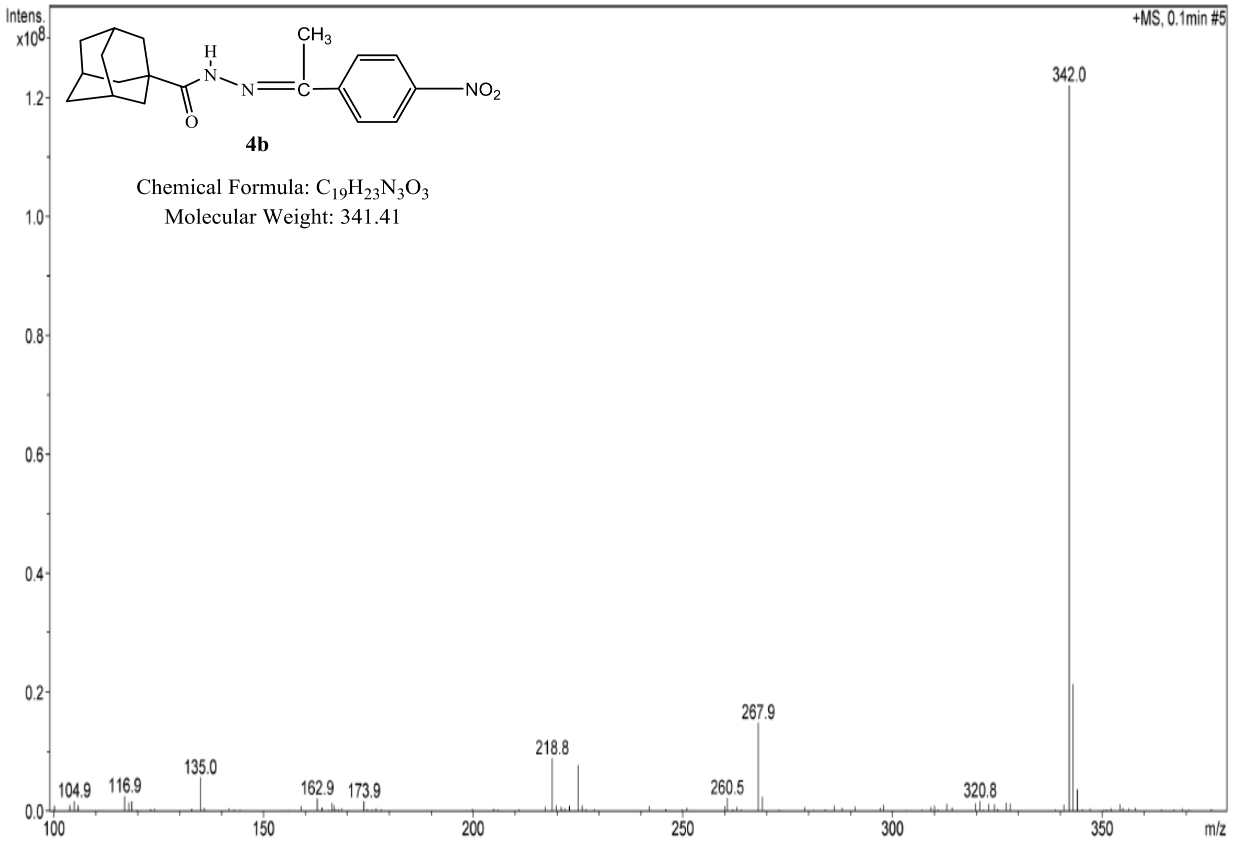

^{1}H-NMR spectrum of compound **4c**

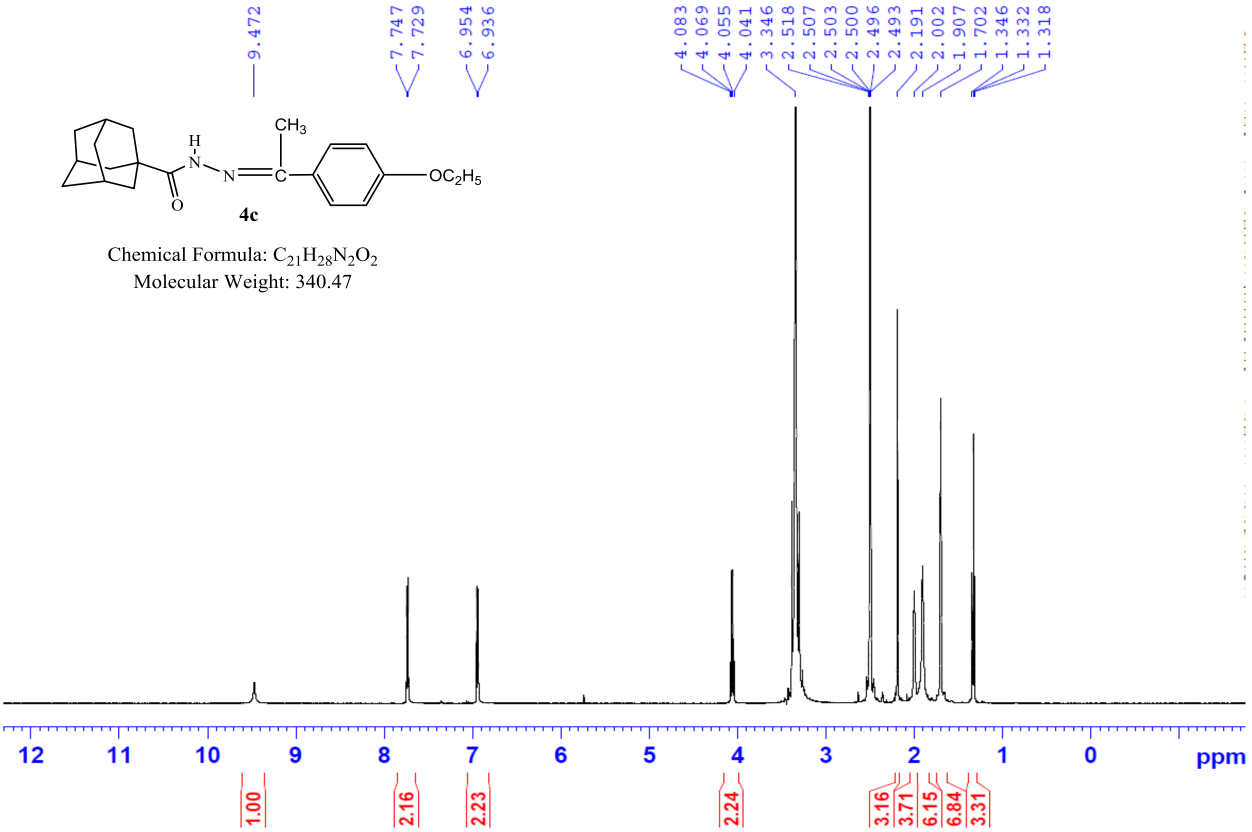

ESI-MS spectrum of compound **4c** (negative)

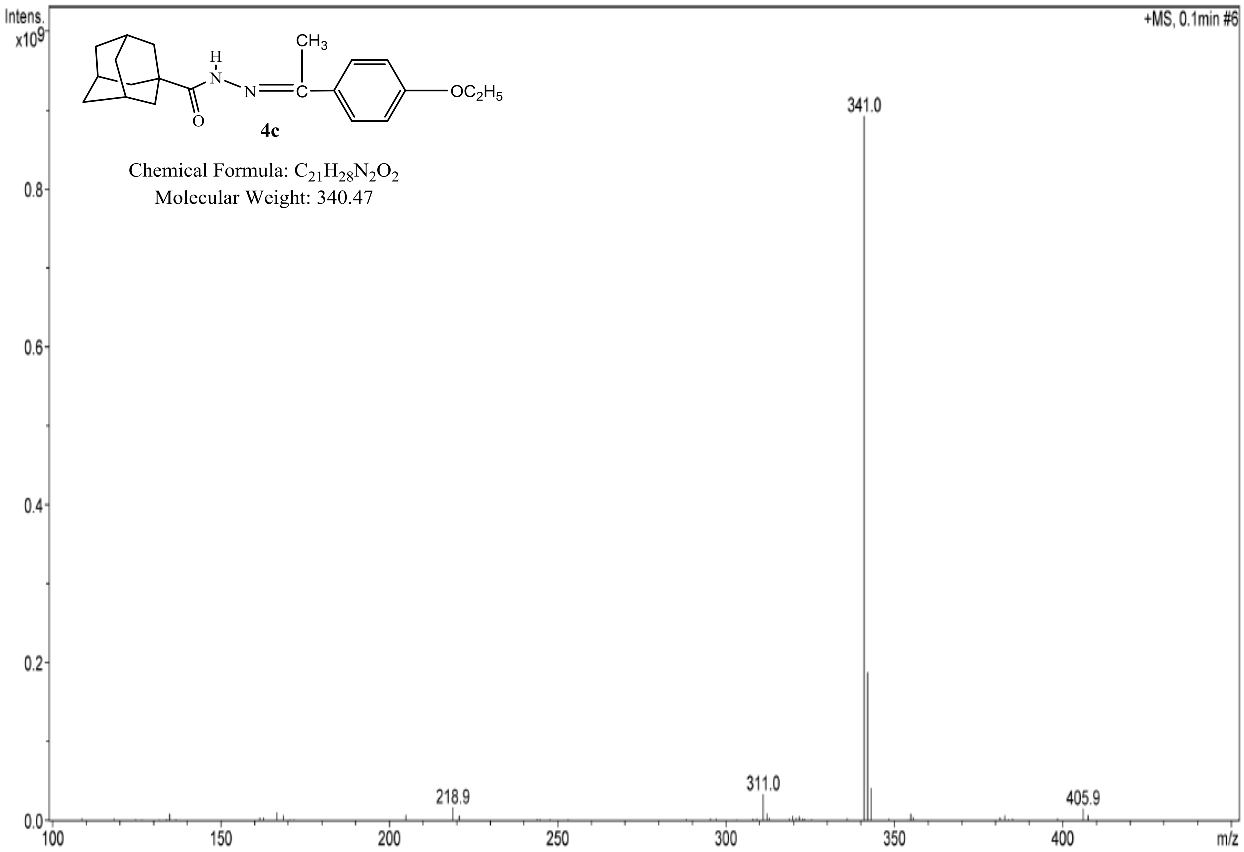

^{1}H-NMR spectrum of compound **4d**

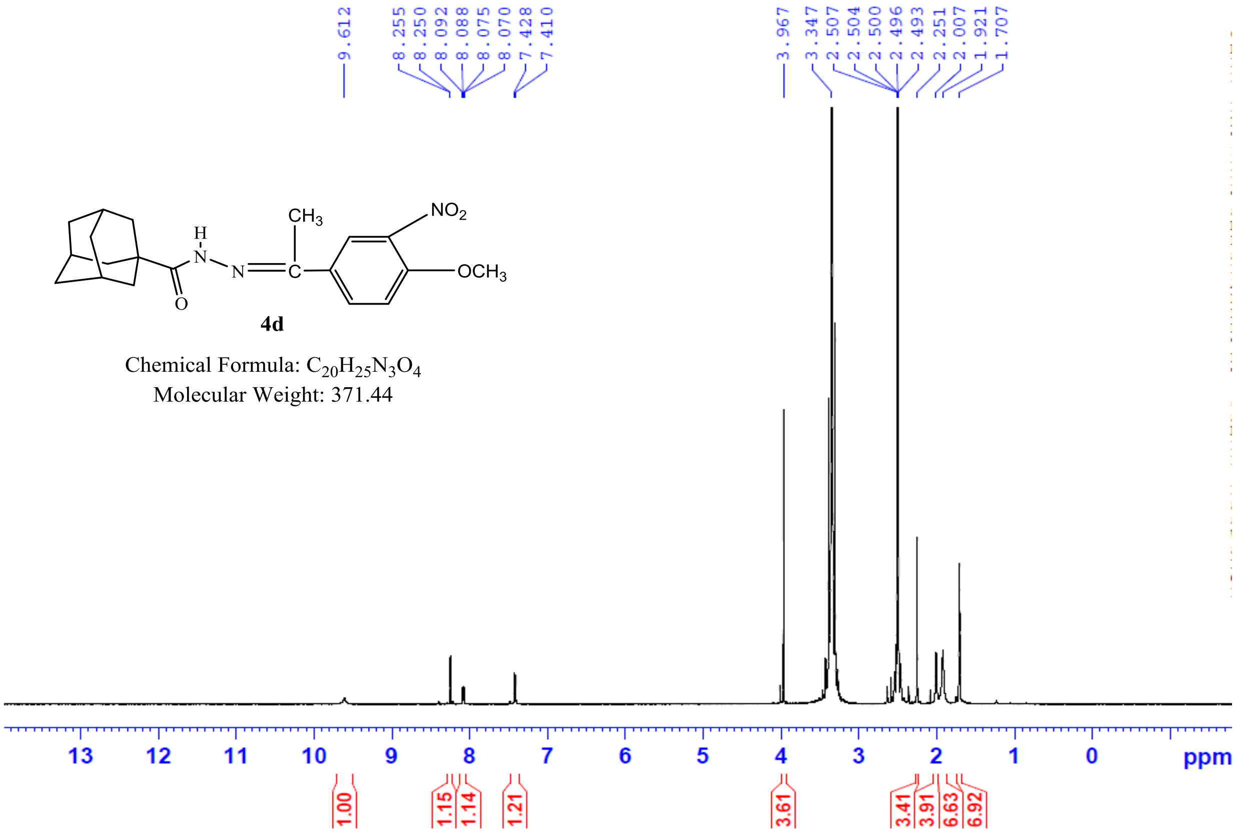

ESI-MS spectrum of compound **4d** (negative)

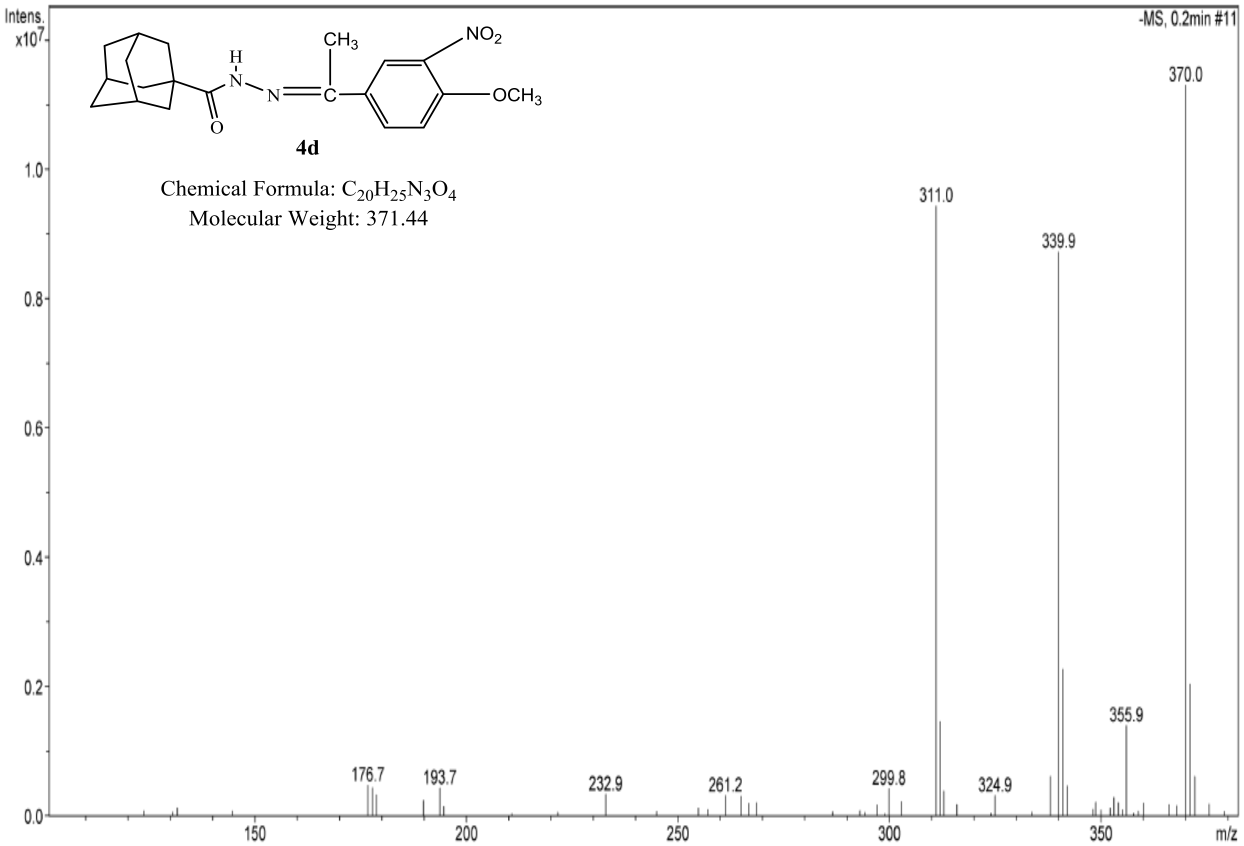

ESI-MS spectrum of compound **4d** (positive)

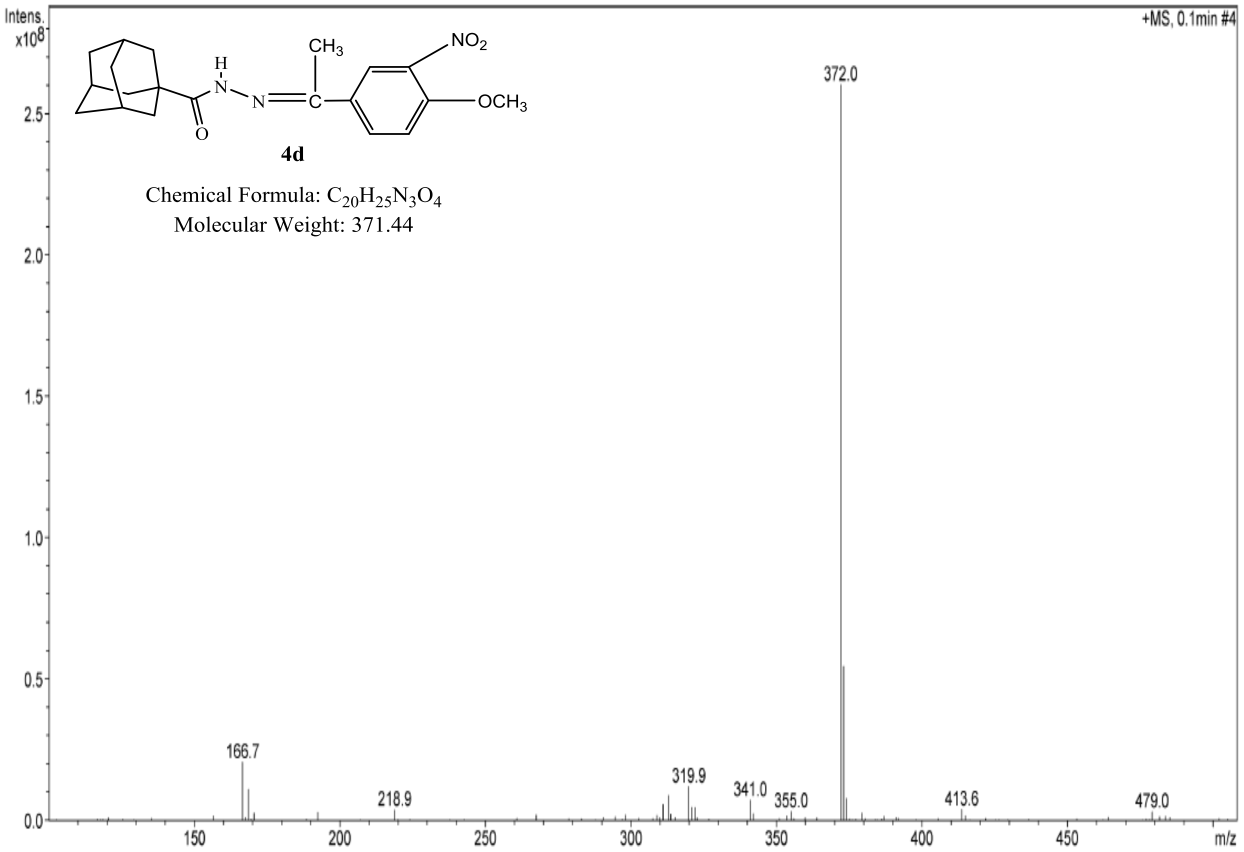

^{1}H-NMR spectrum of compound **4e**

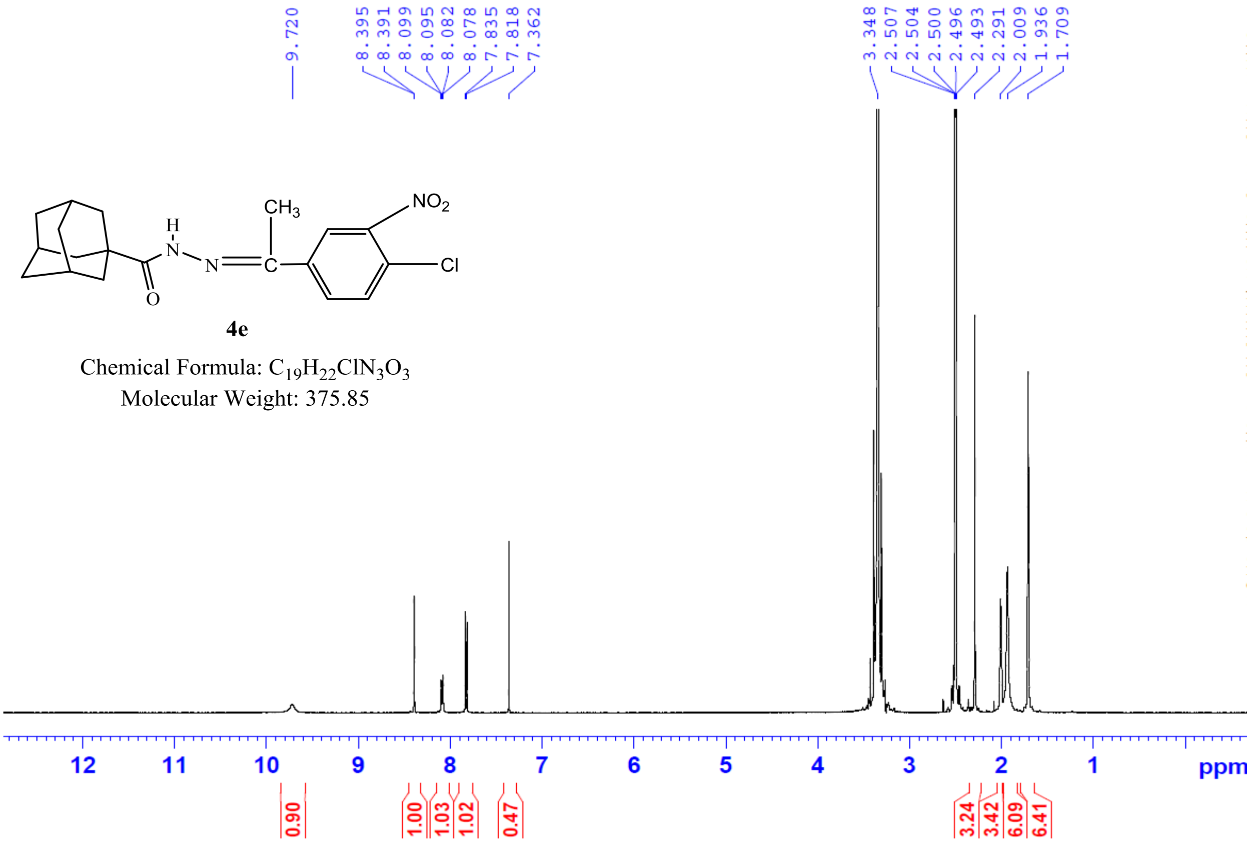

ESI-MS spectrum of compound **4e** (negative)

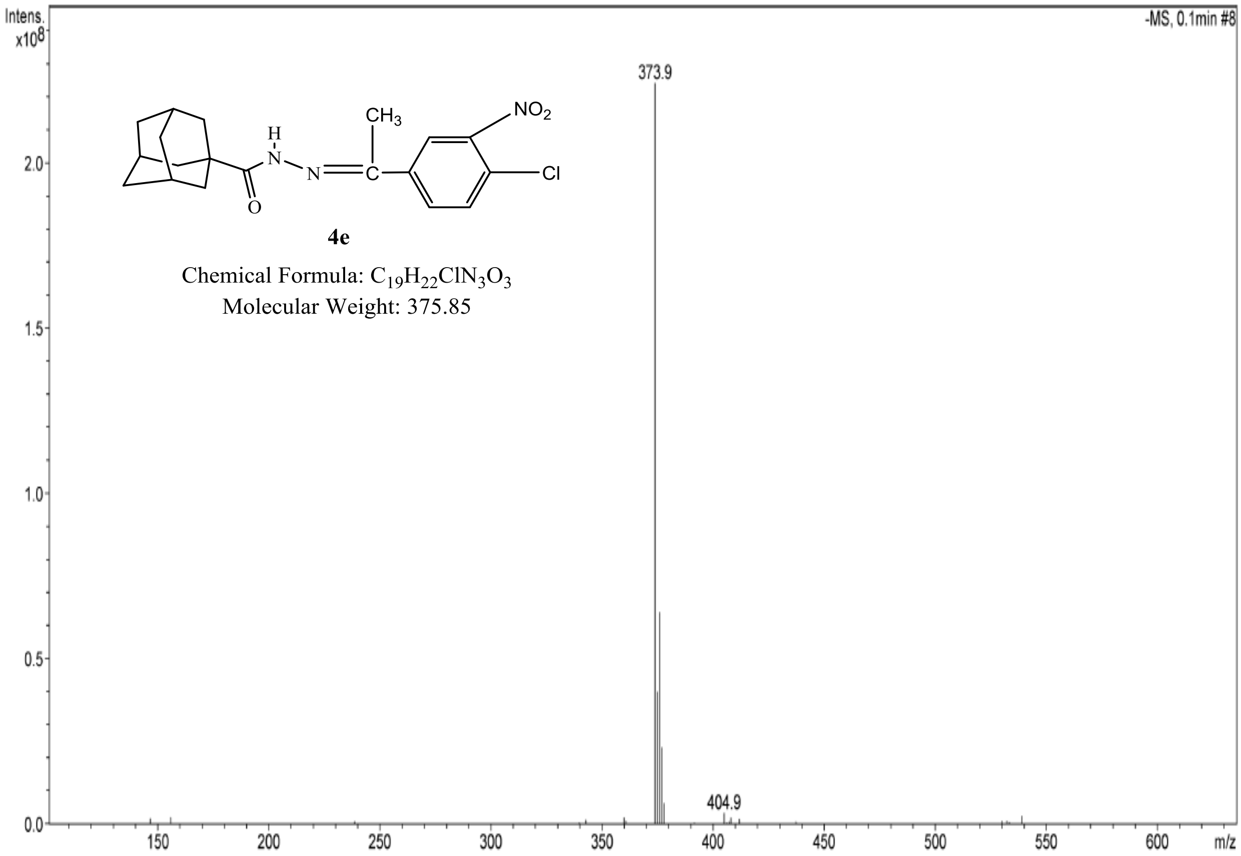

ESI-MS spectrum of compound **4e** (positive)

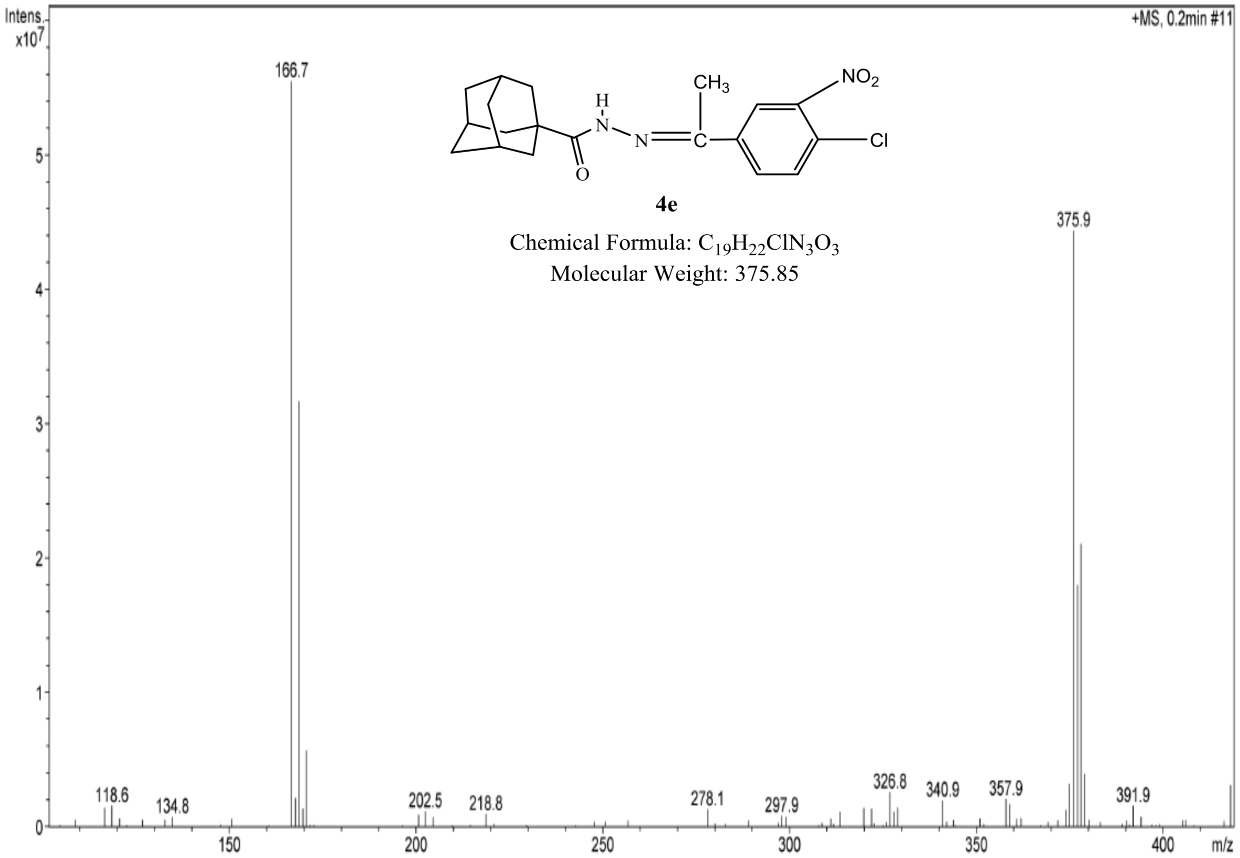

¹H-NMR spectrum of compound **4f**

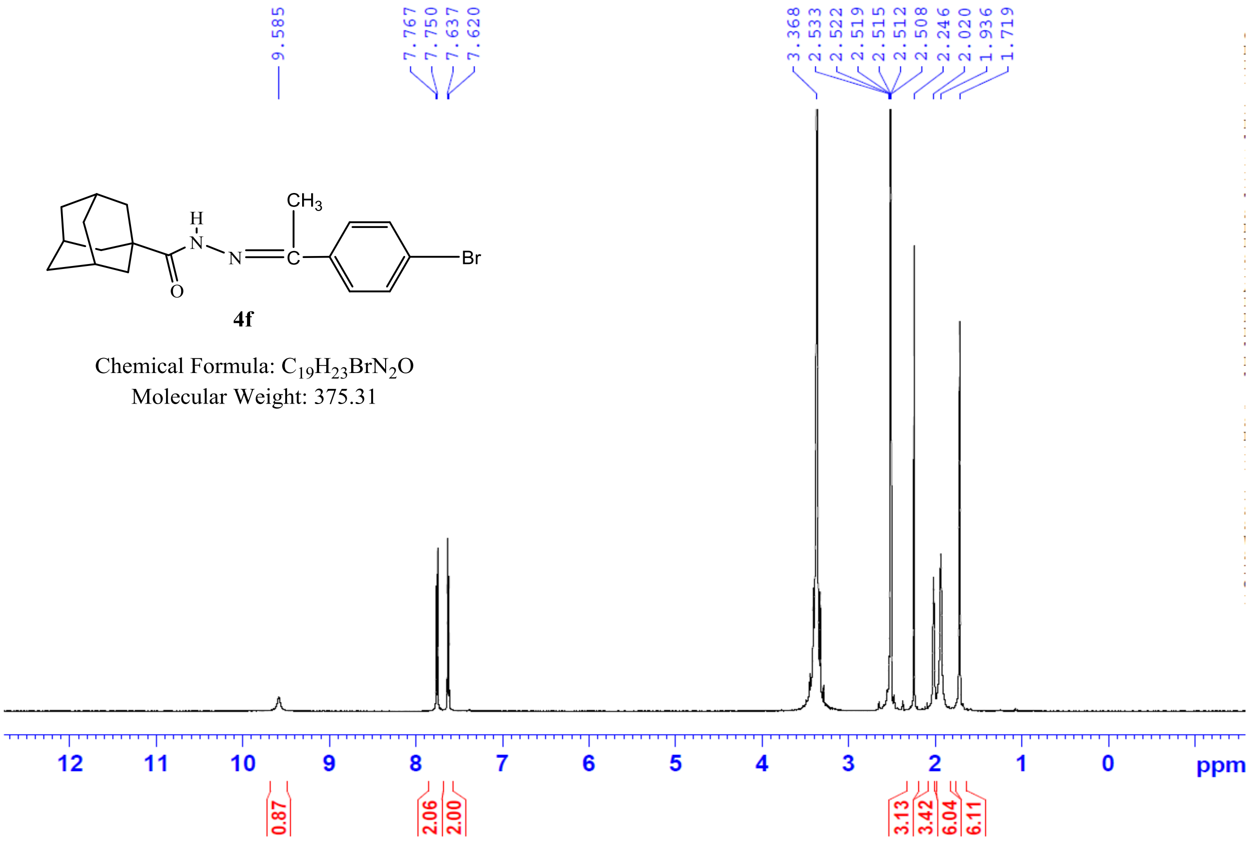

ESI-MS spectrum of compound **4f** (positive)

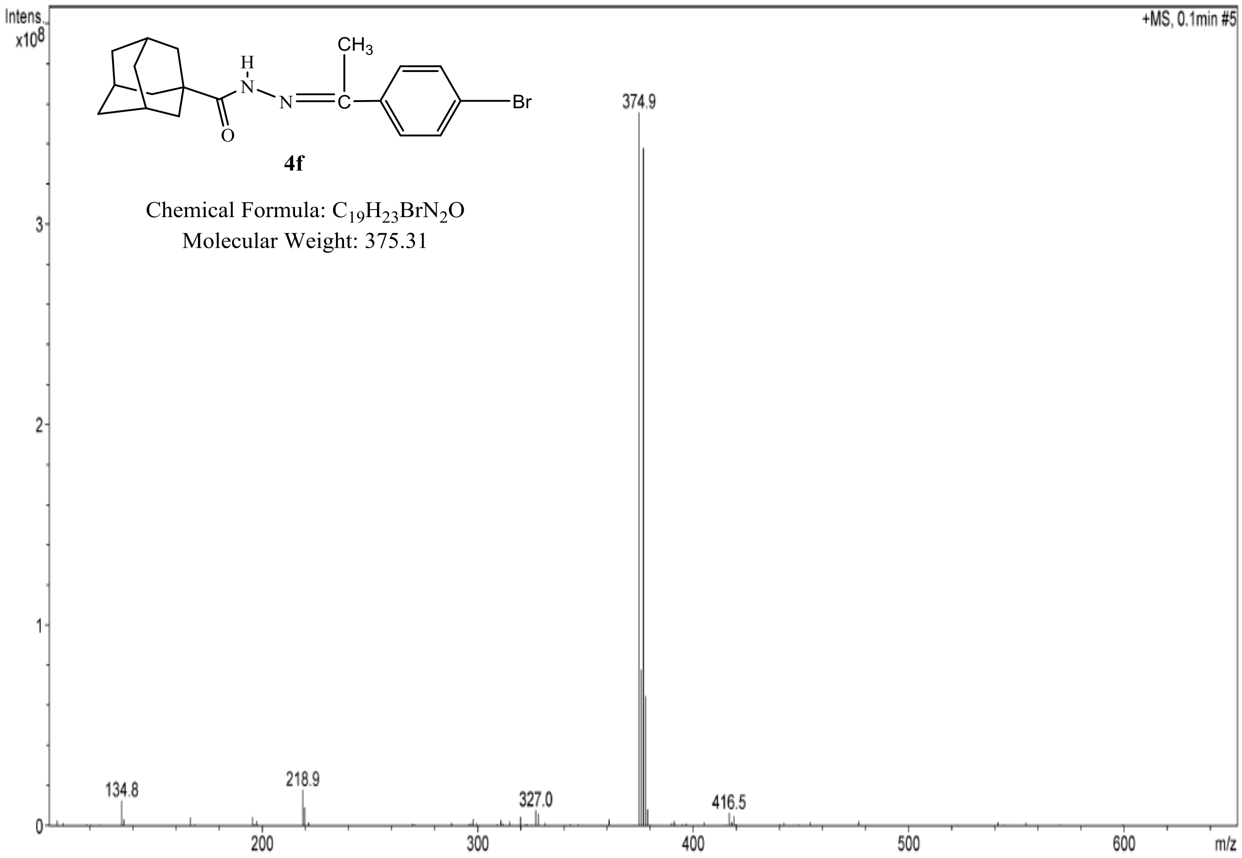

^{1}H-NMR spectrum of compound **4g**

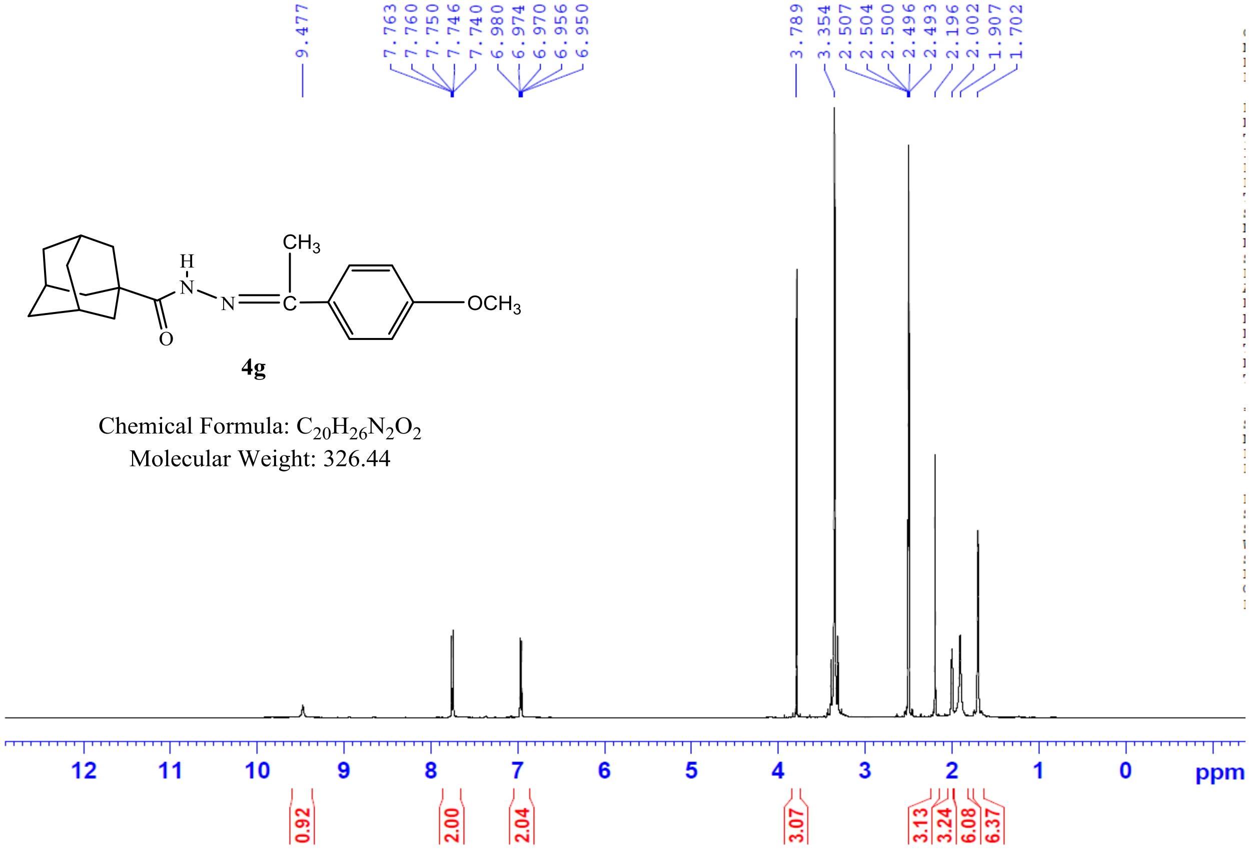

ESI-MS spectrum of compound **4g** (positive)

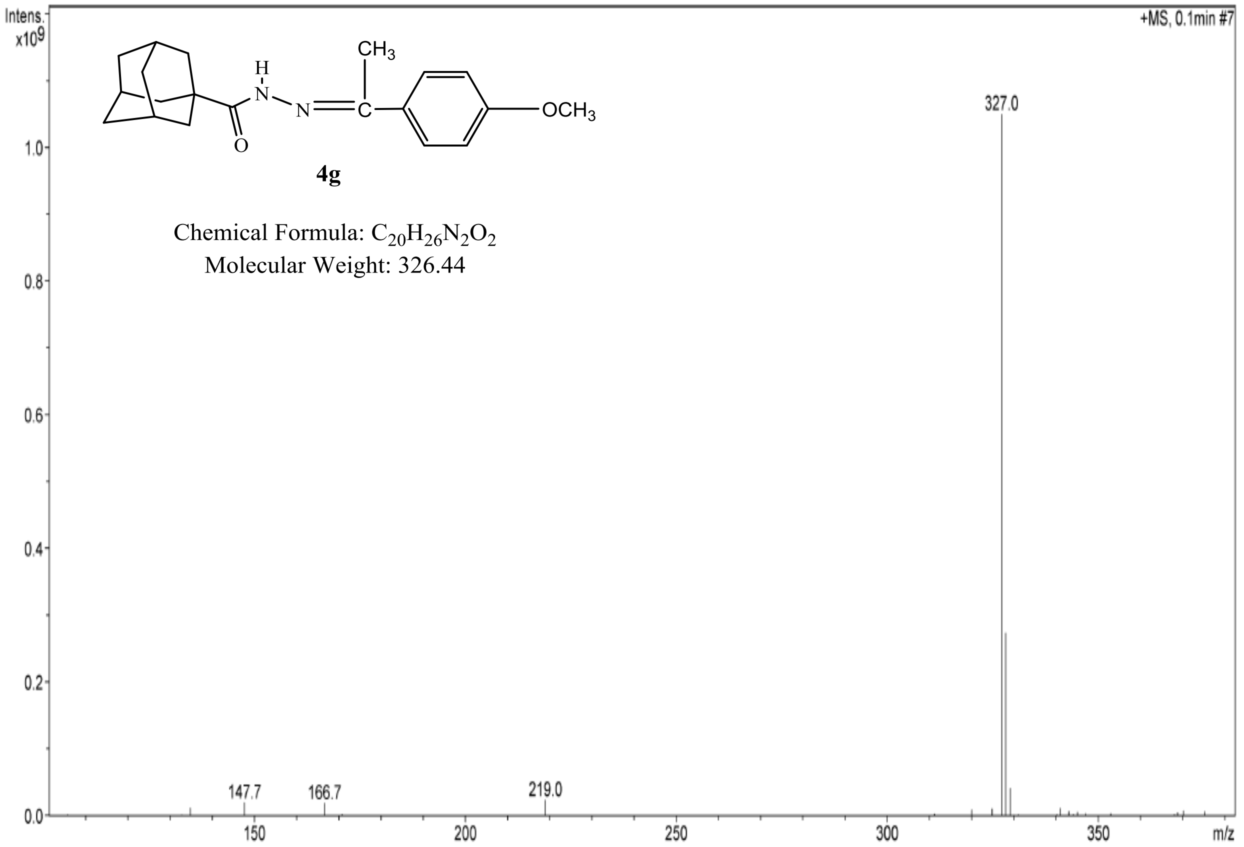

^{1}H-NMR spectrum of compound **4h**

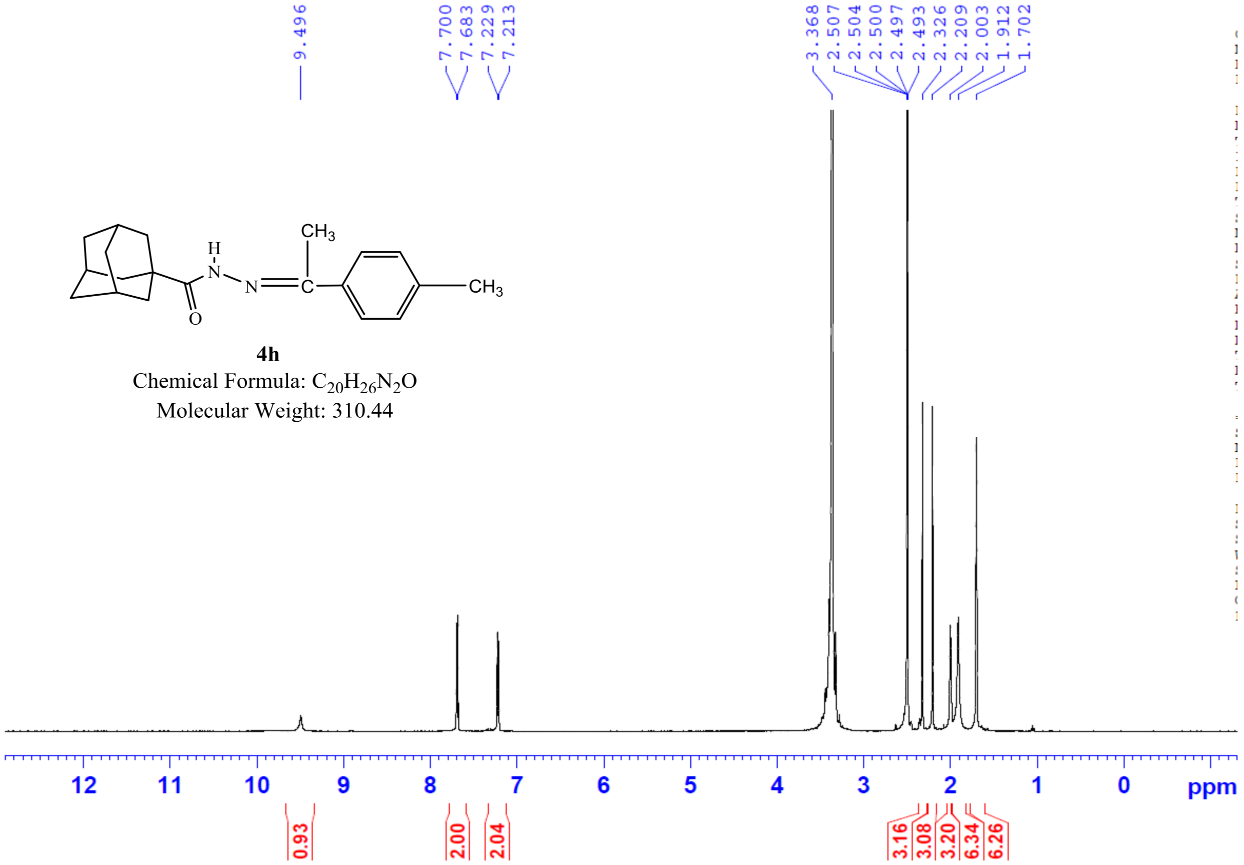

ESI-MS spectrum of compound **4h** (positive)

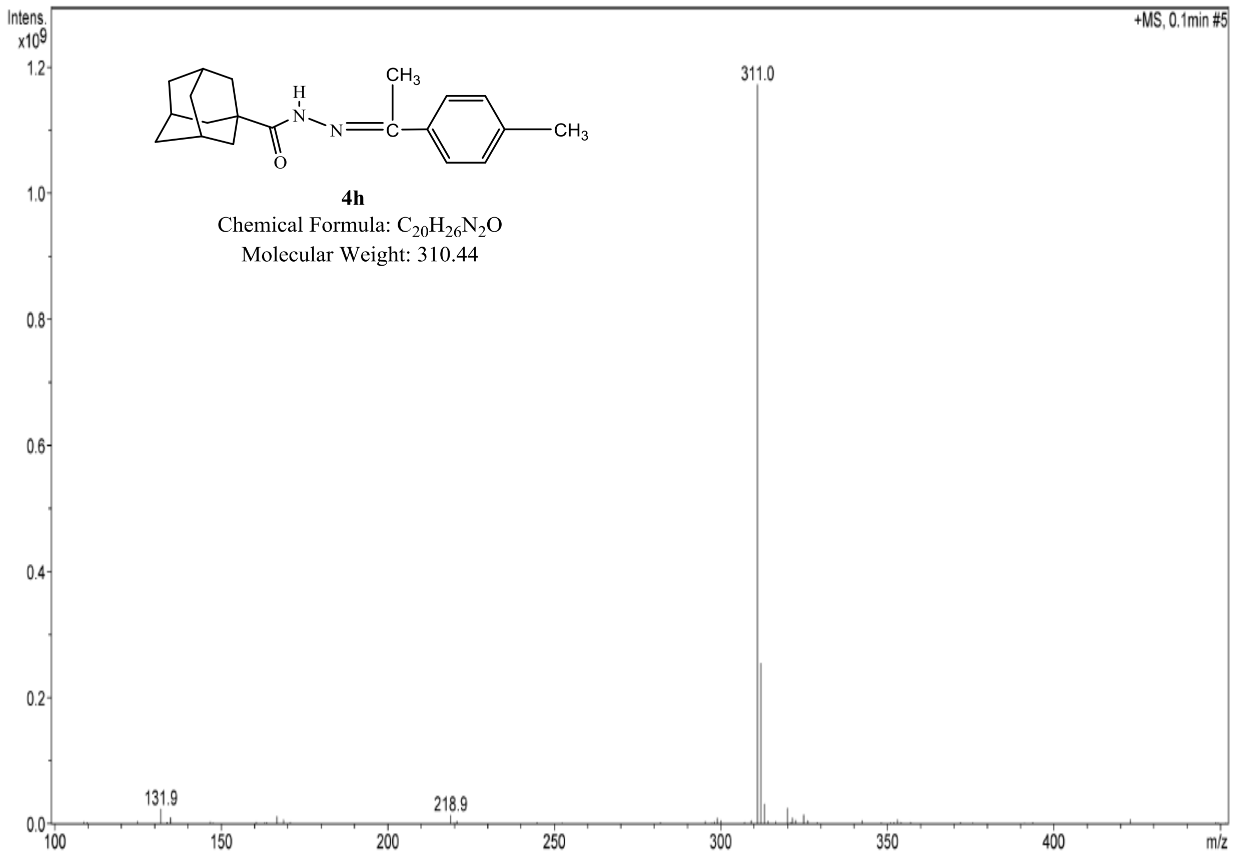

^{1}H-NMR spectrum of compound **4i**

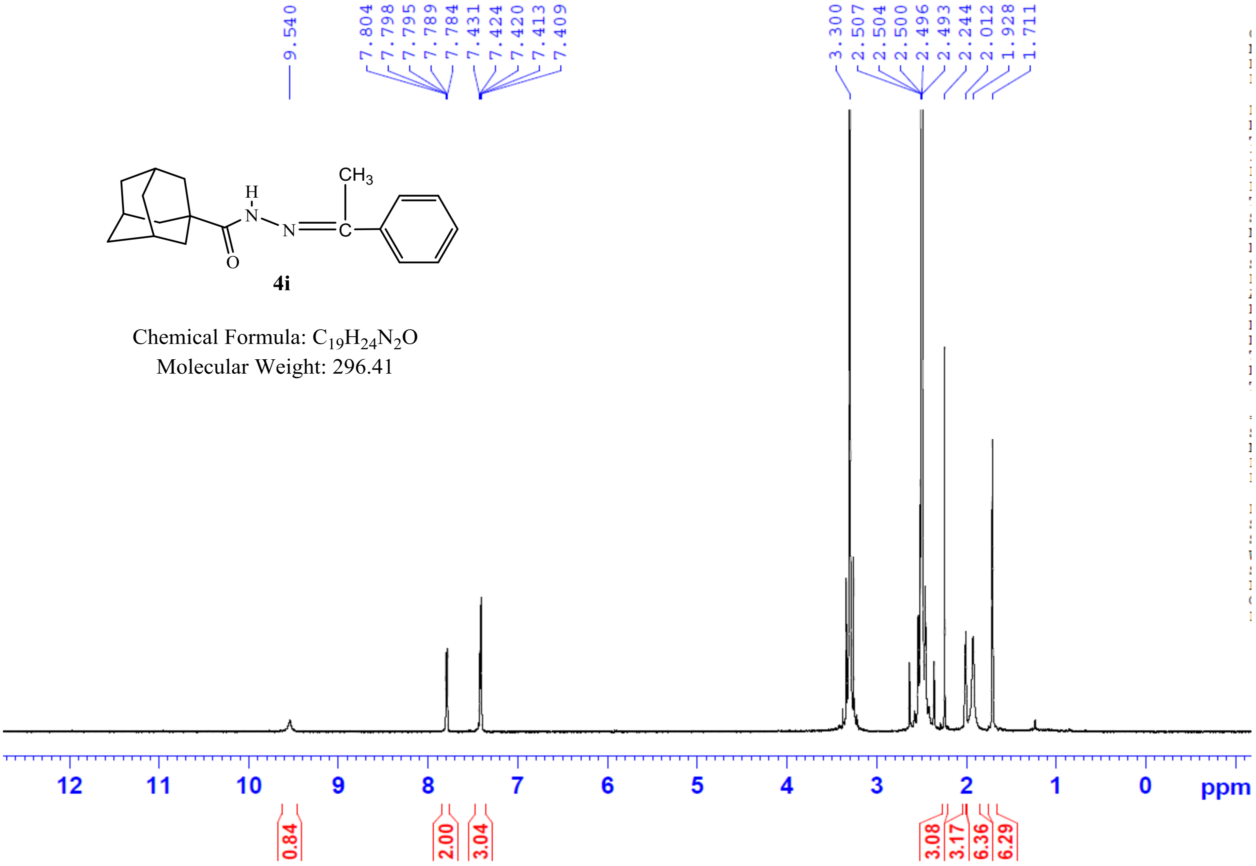

ESI-MS spectrum of compound **4i** (positive)

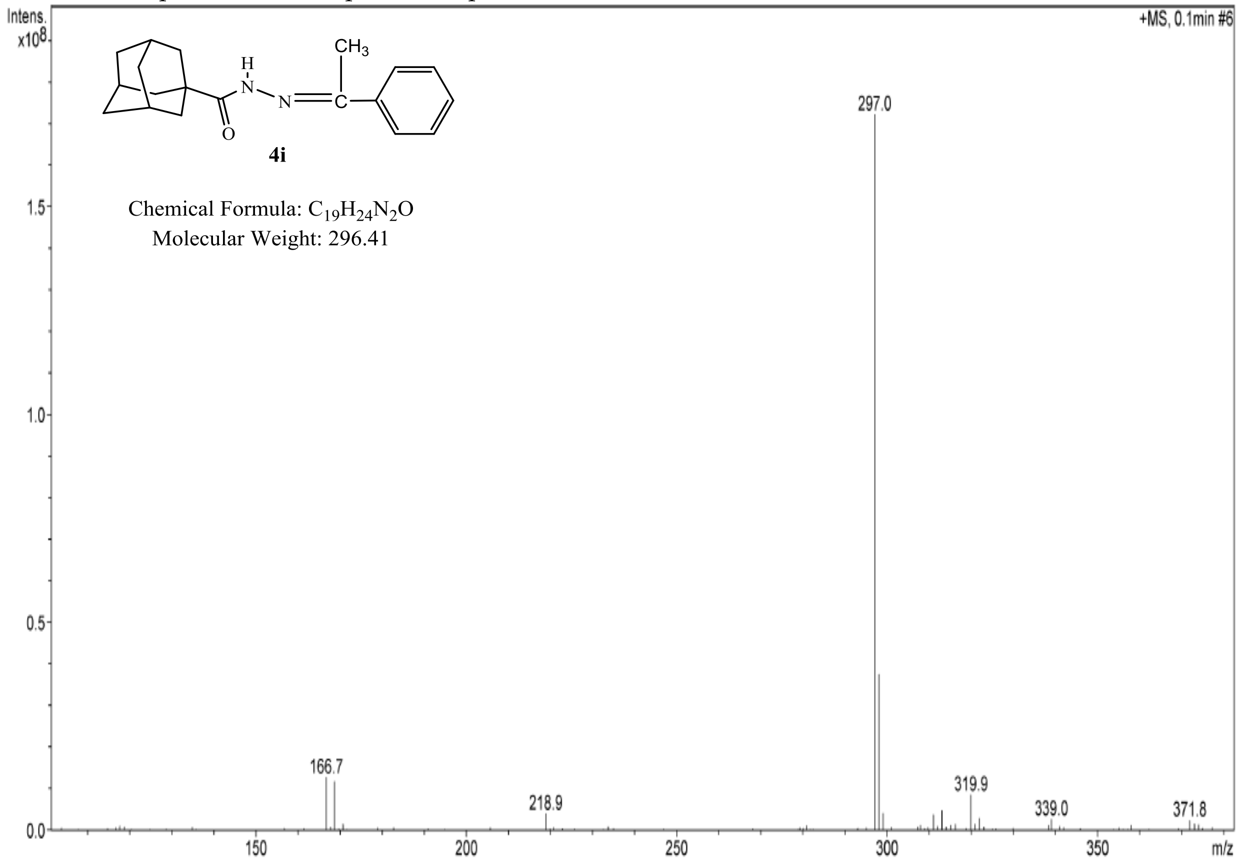

^{1}H-NMR spectrum of compound **5a**

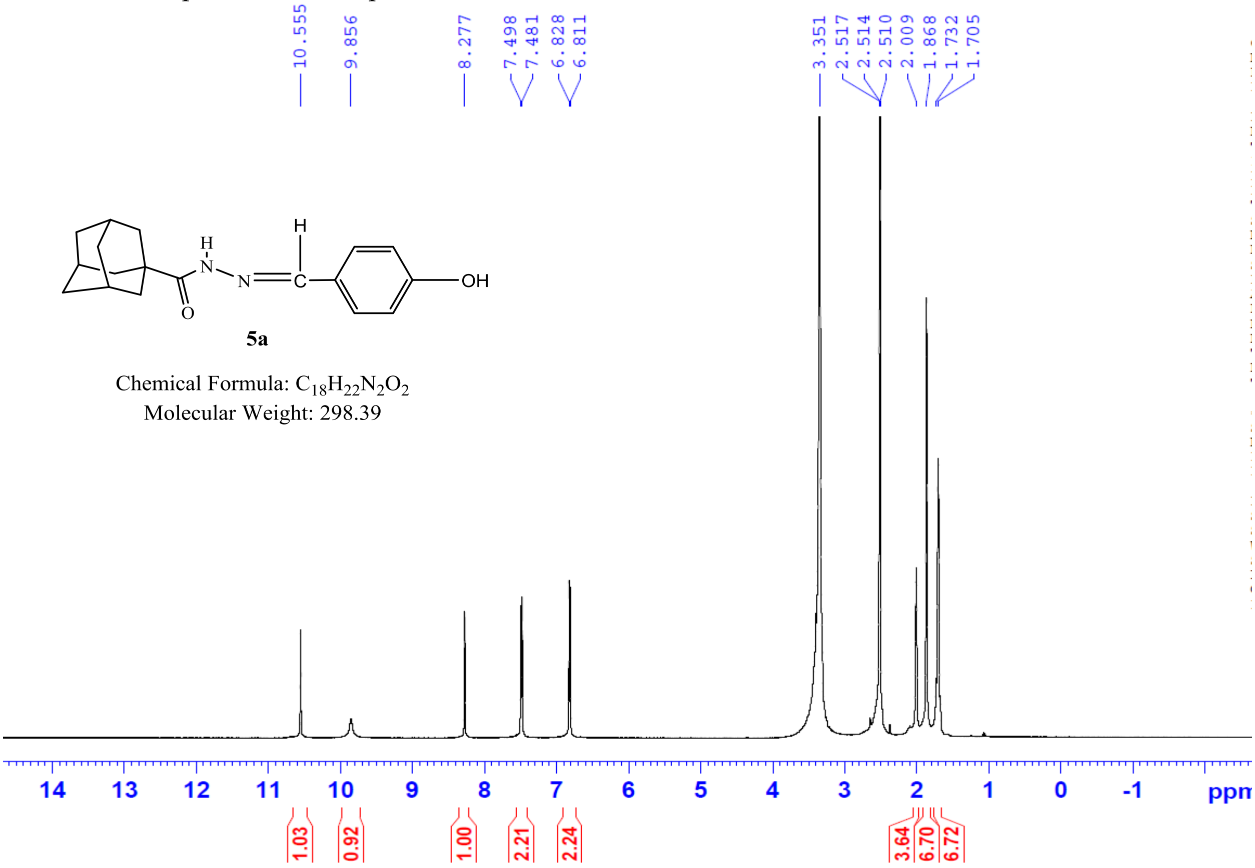

ESI-MS spectrum of compound **5a** (negative)

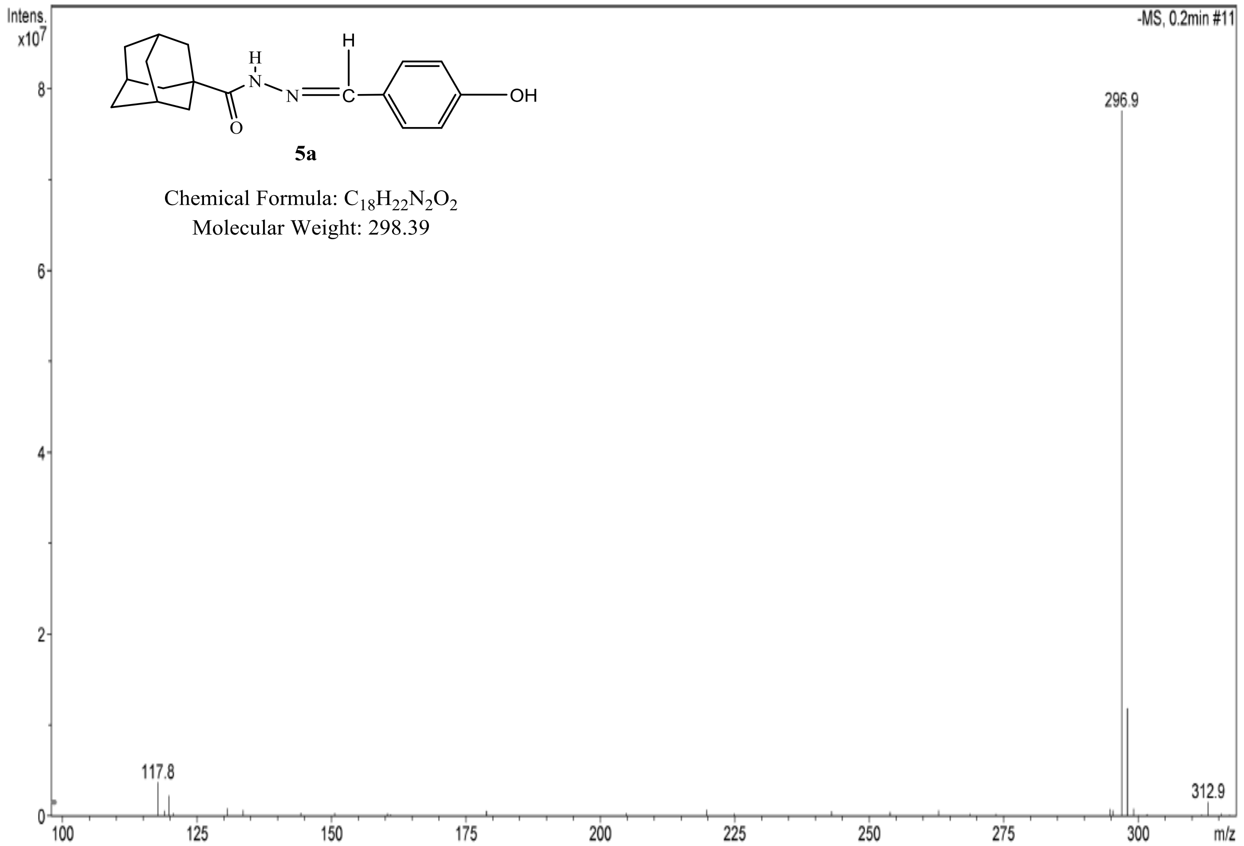

ESI-MS spectrum of compound **5a** (positive)

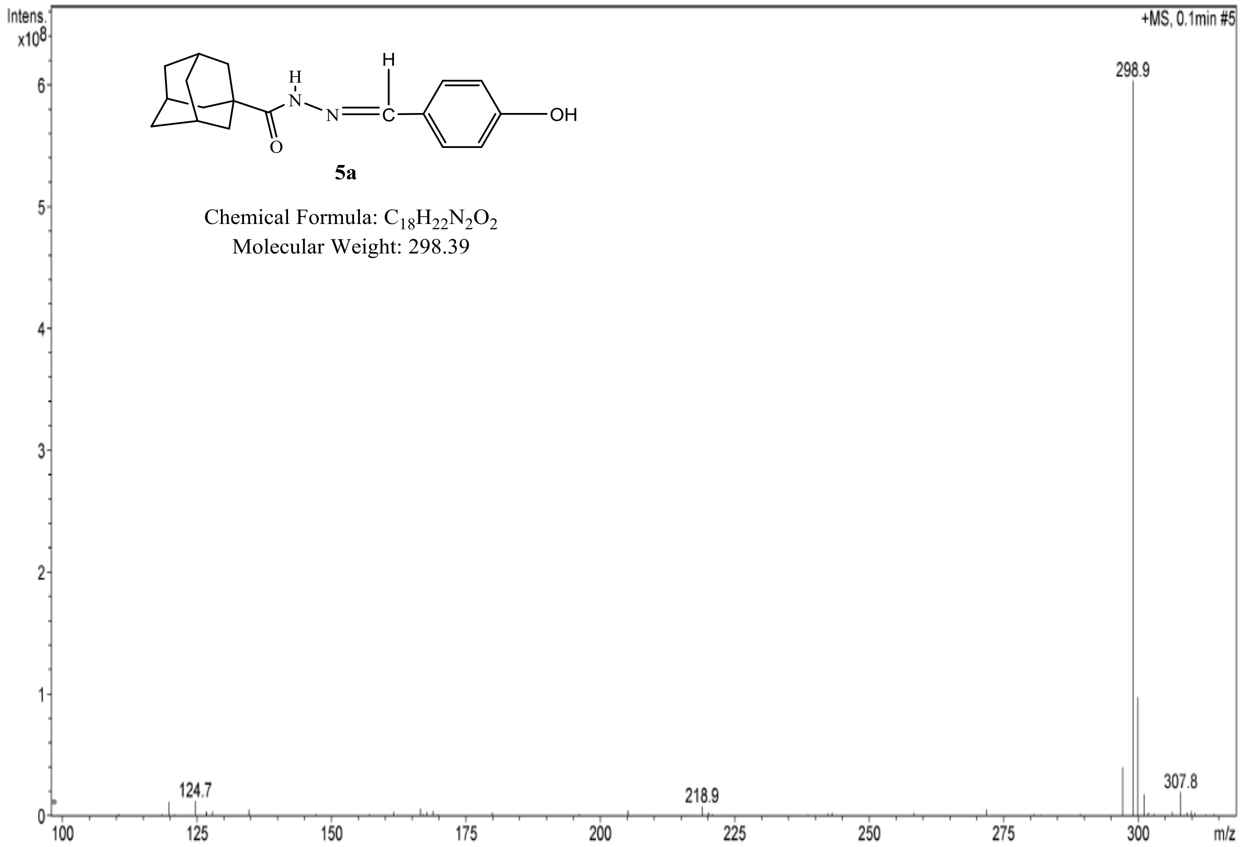

^{1}H-NMR spectrum of compound **5c**

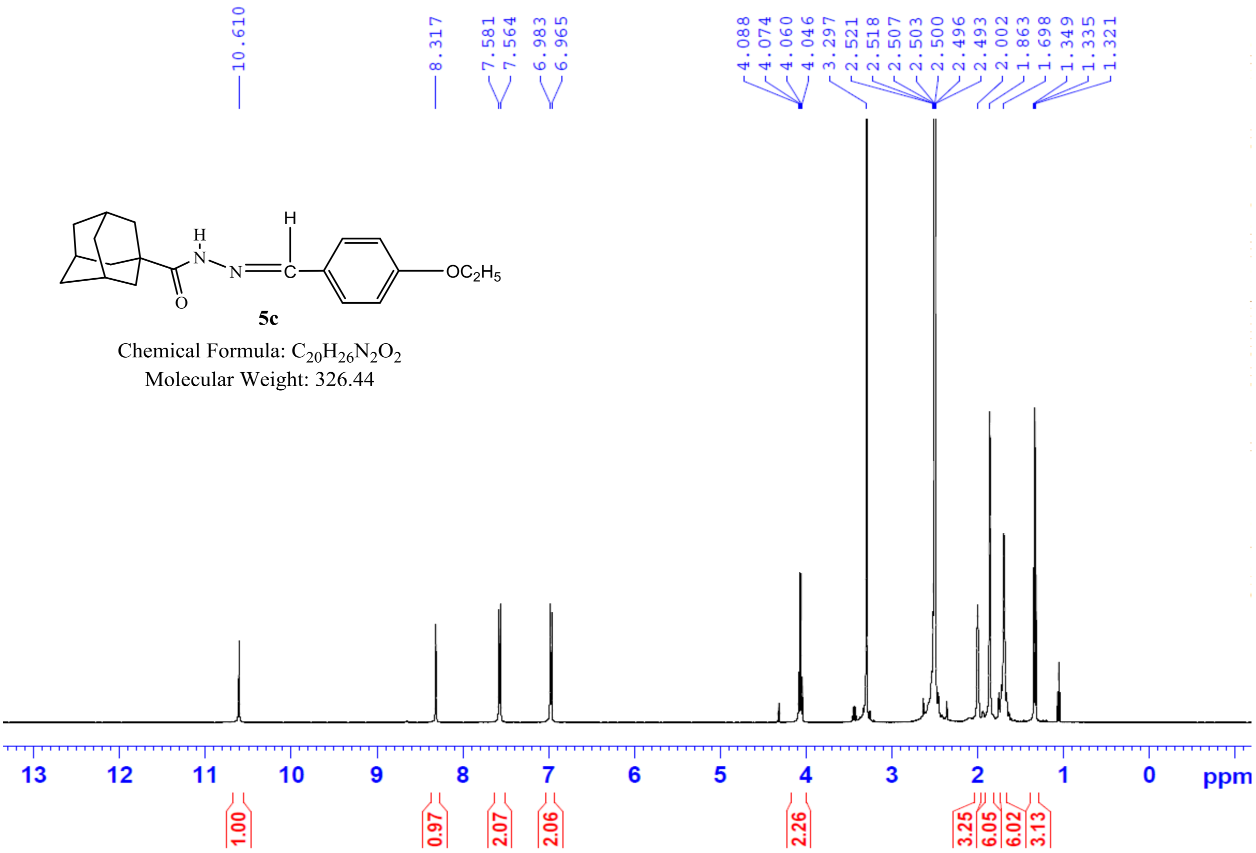

ESI-MS spectrum of compound **5c** (positive)

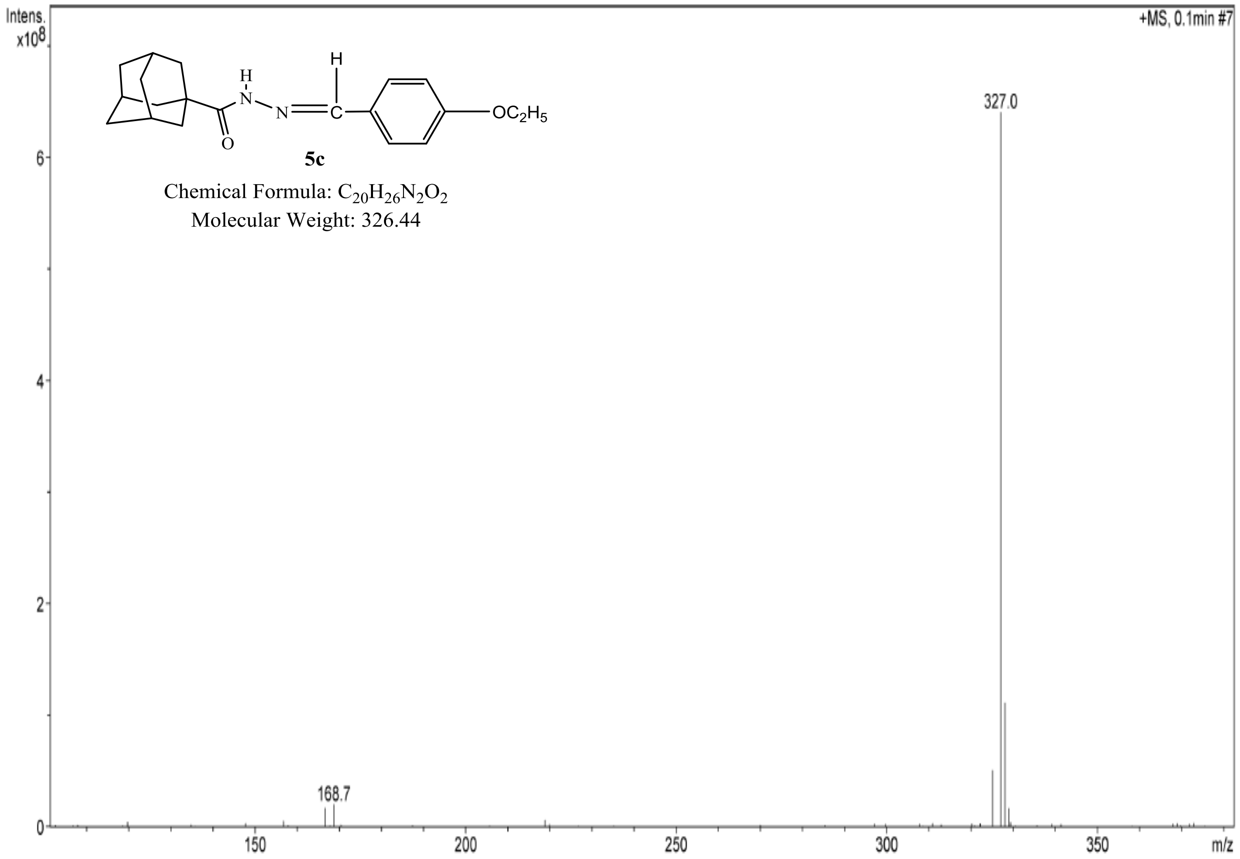

^{1}H-NMR spectrum of compound **5e**

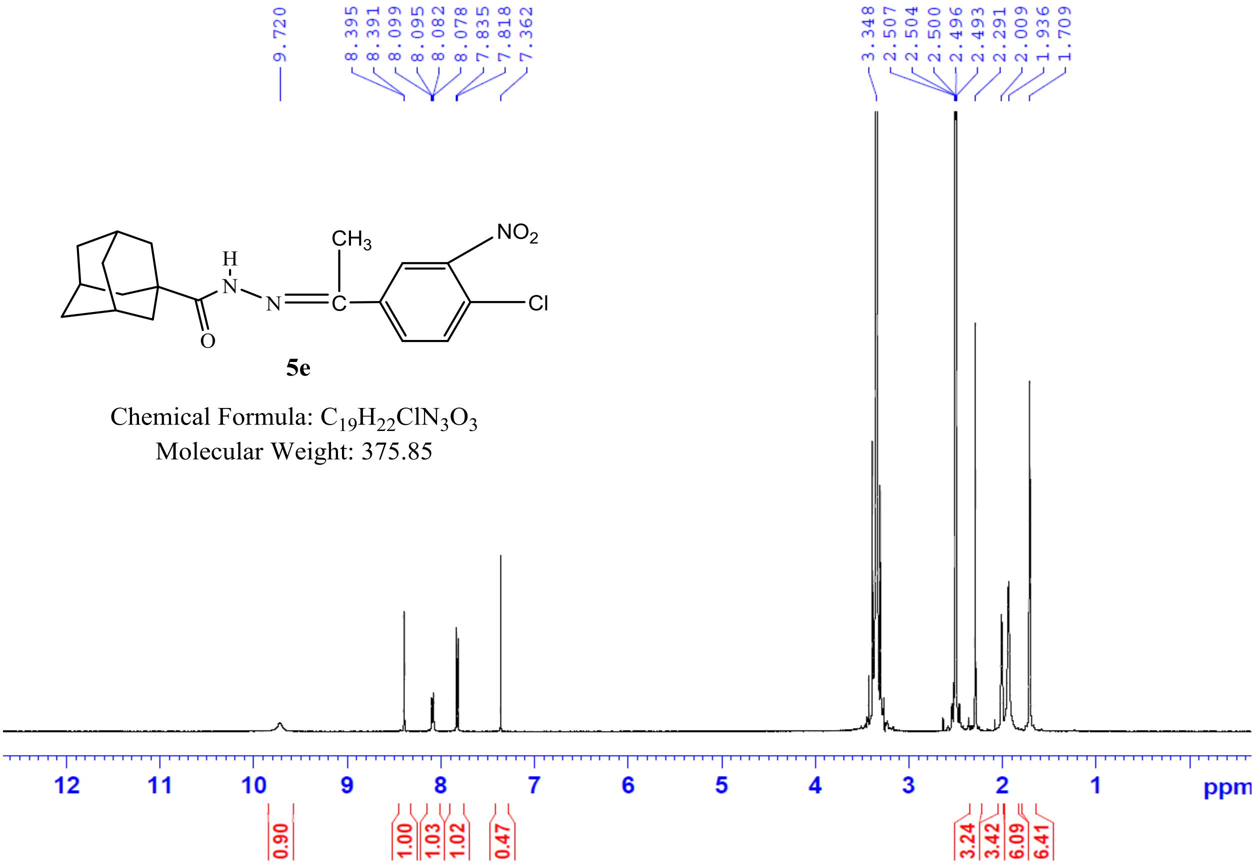

ESI-MS spectrum of compound **5e** (negative)

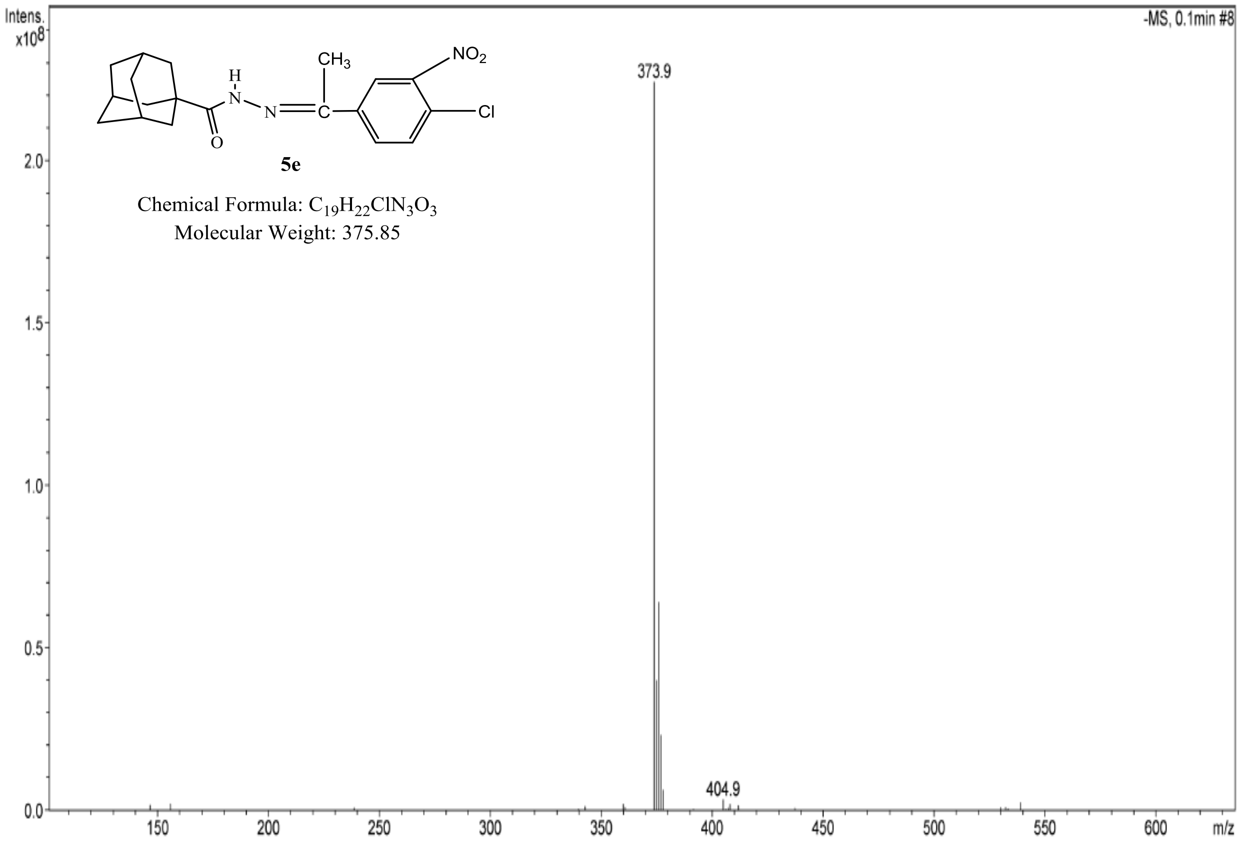

ESI-MS spectrum of compound **5e** (positive)

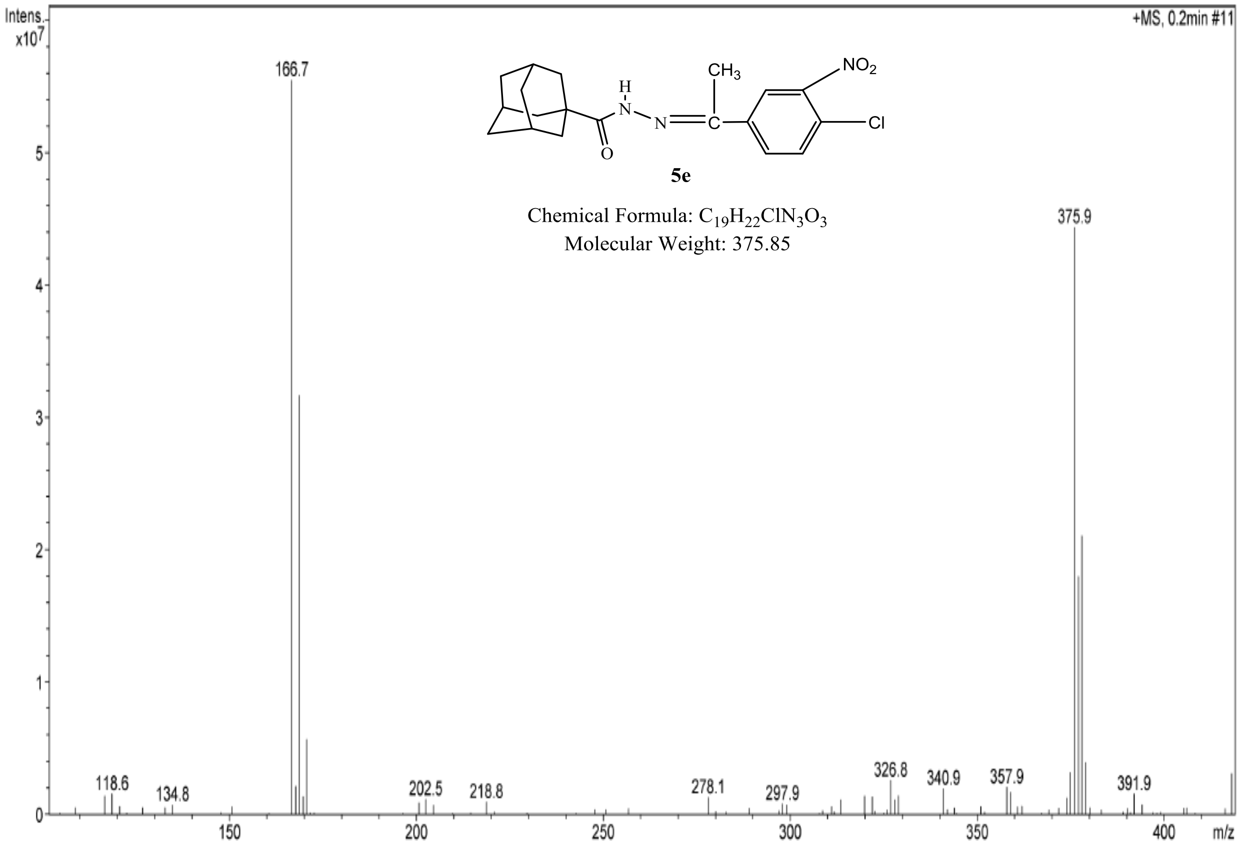

^{1}H-NMR spectrum of compound **5i**

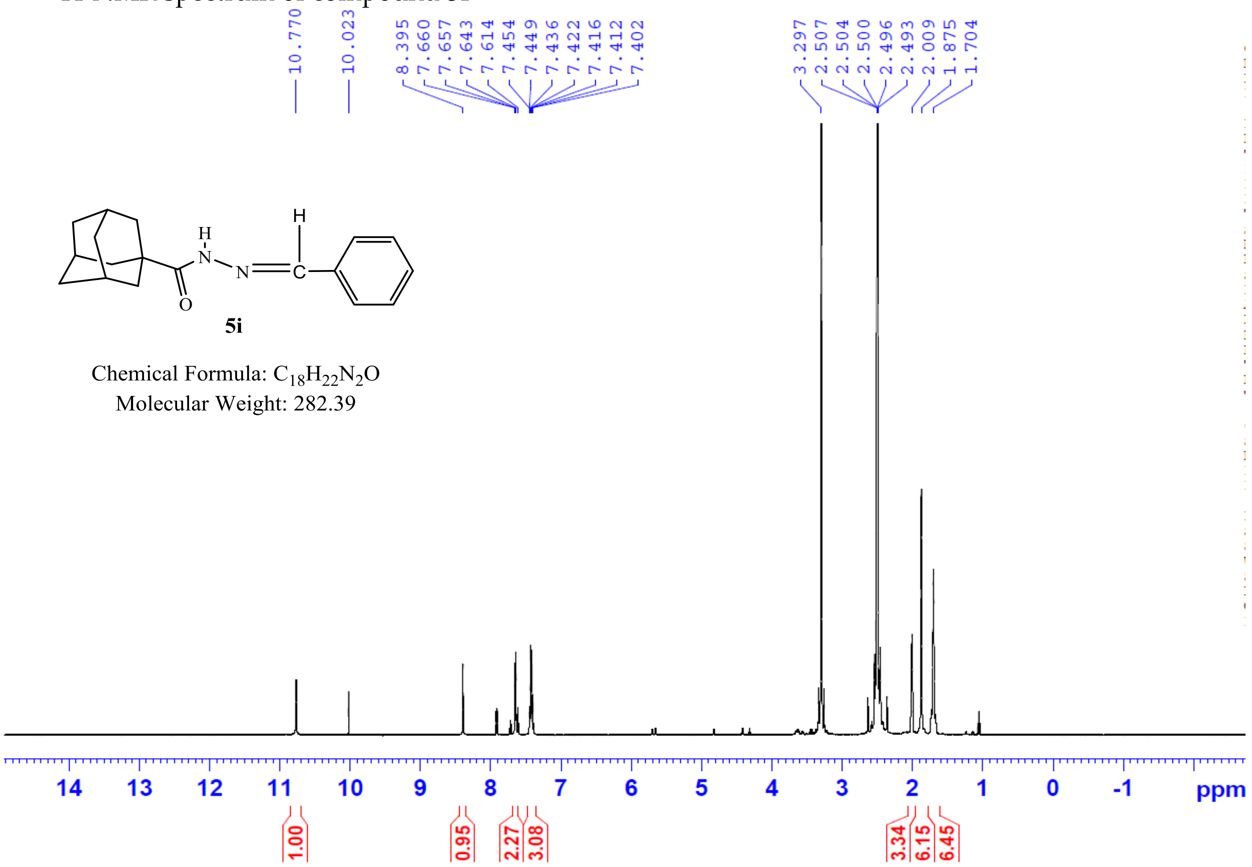

ESI-MS spectrum of compound **5i** (positive)

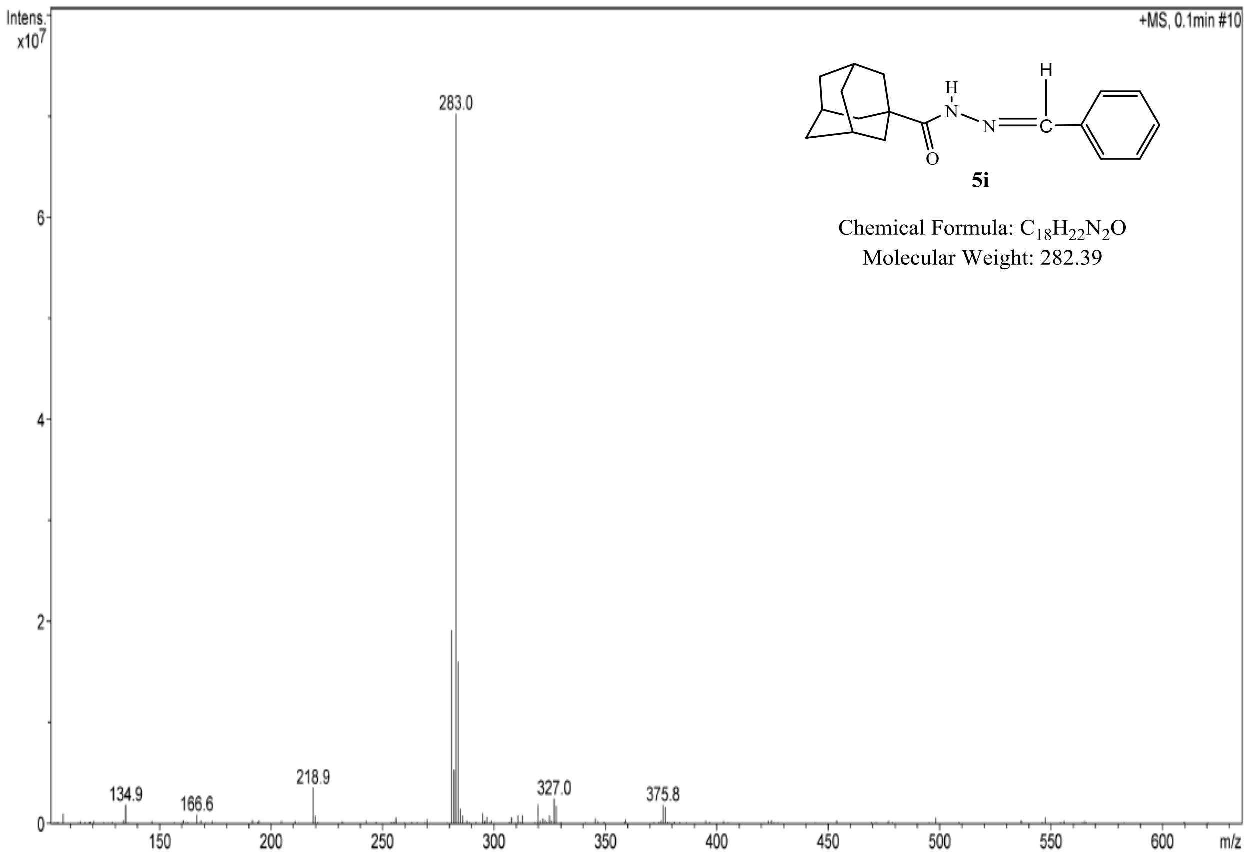

^{1}H-NMR spectrum of compound **5j**

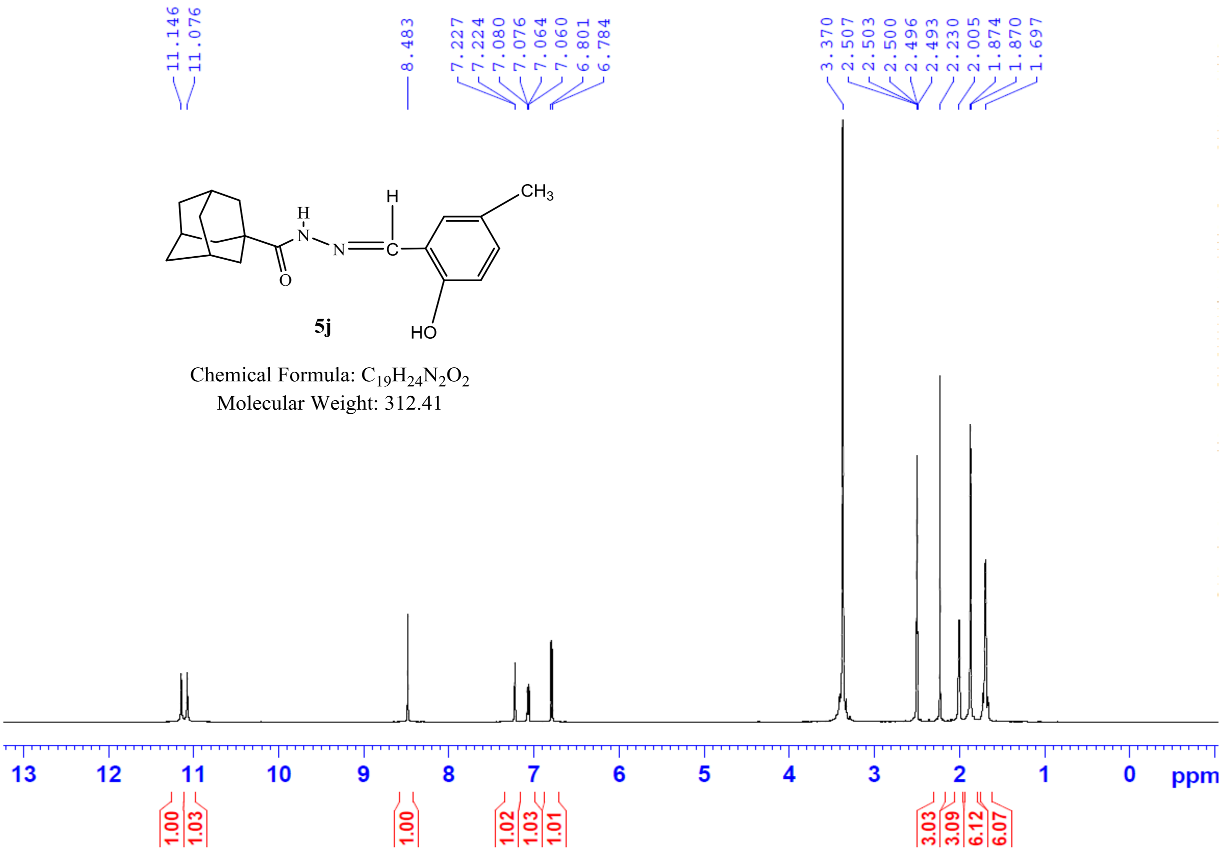

ESI-MS spectrum of compound **5j** (negative)

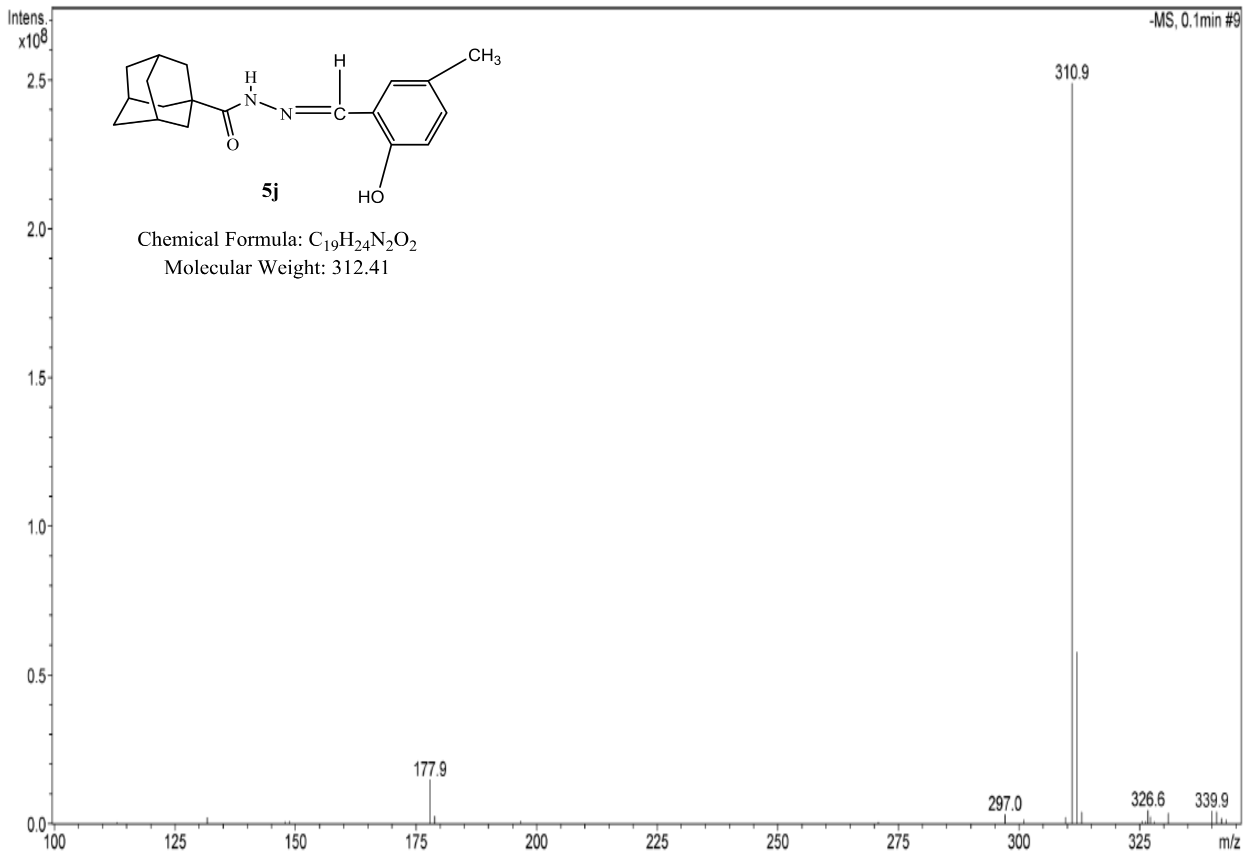

ESI-MS spectrum of compound **5j** (positive)

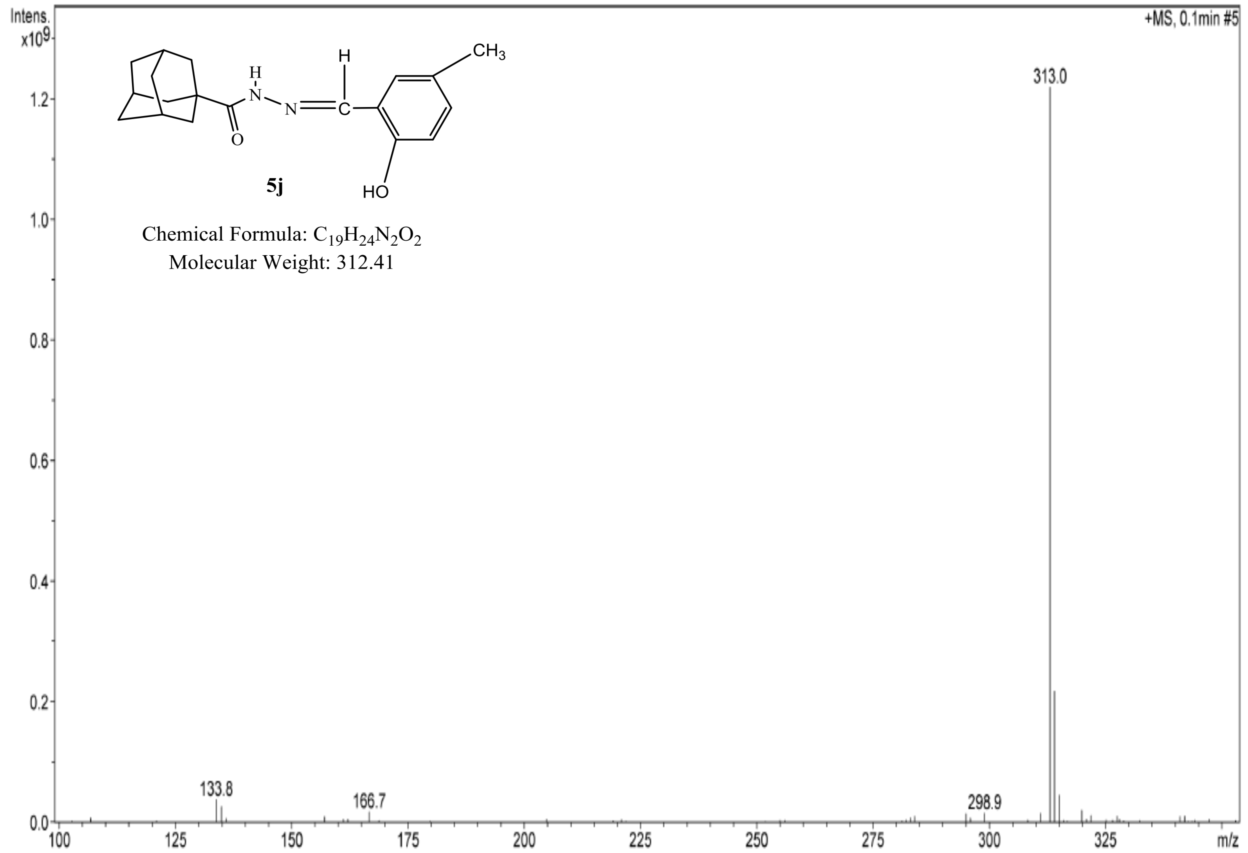

^{1}H-NMR spectrum of compound **5k**

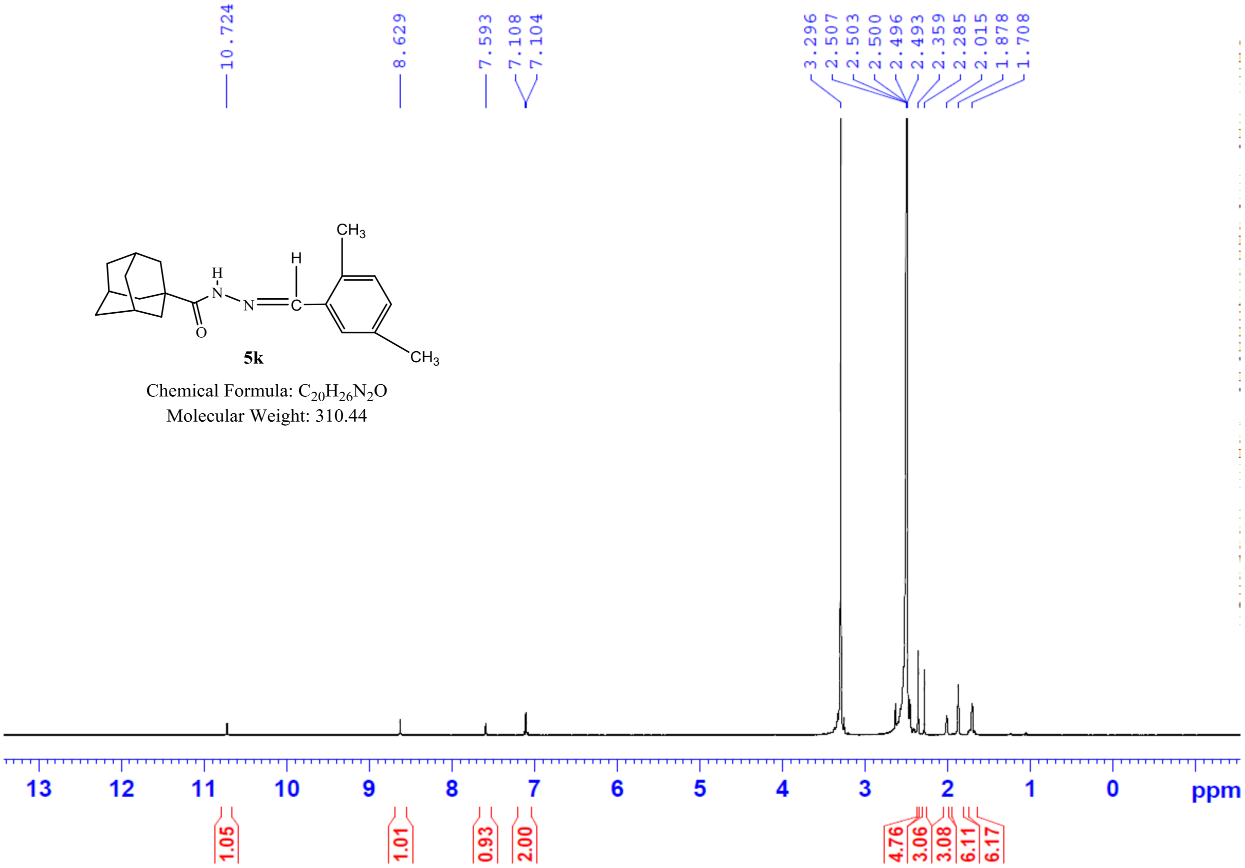

ESI-MS spectrum of compound **5k** (positive)

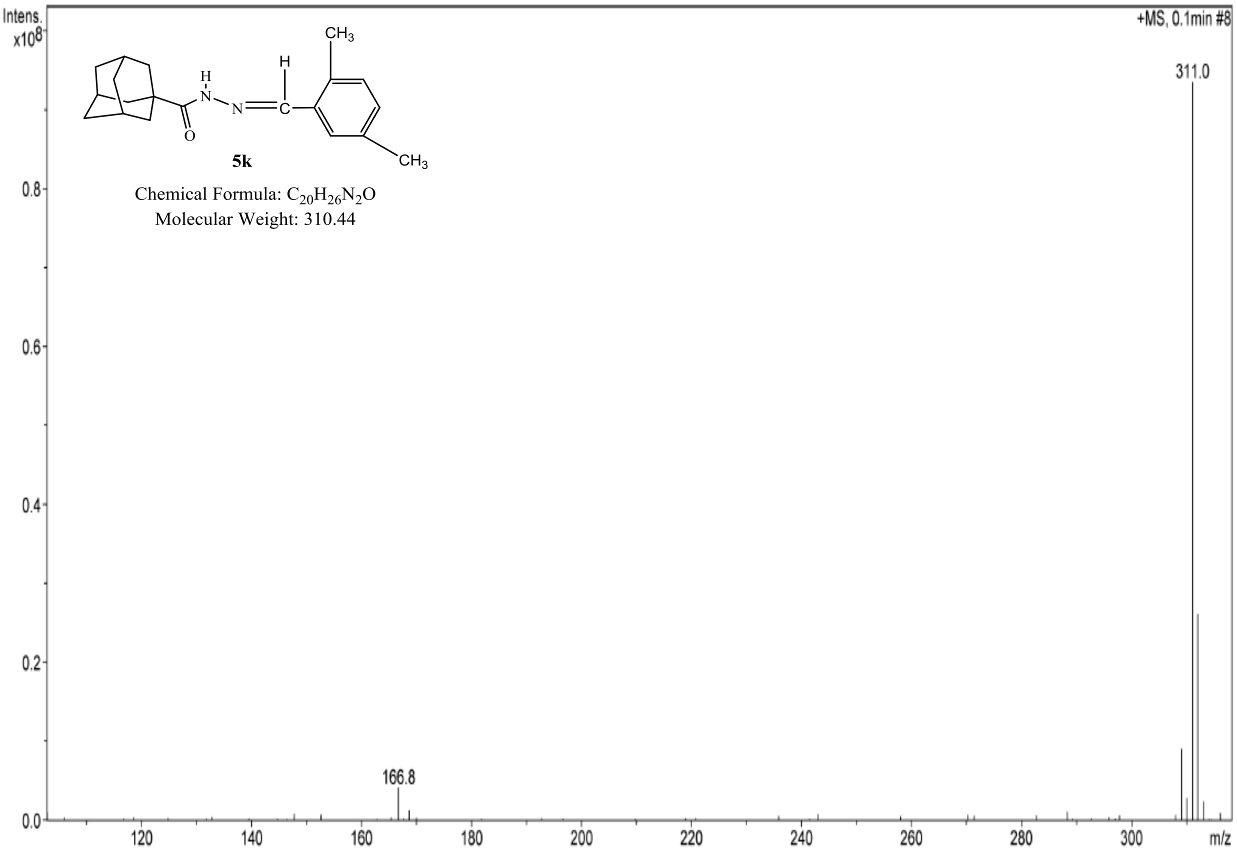

Supplement: Supplementary file 1 [file molecules-24-04000-s001.pdf]
